# A haplotype-resolved draft genome of the European sardine (*Sardina pilchardus*)

**DOI:** 10.1093/gigascience/giz059

**Published:** 2019-05-21

**Authors:** Bruno Louro, Gianluca De Moro, Carlos Garcia, Cymon J Cox, Ana Veríssimo, Stephen J Sabatino, António M Santos, Adelino V M Canário

**Affiliations:** 1 CCMAR Centre of Marine Sciences, University of Algarve, Campus de Gambelas, 8005–139 Faro, Portugal; 2 CIBIO, Centro de Investigação em Biodiversidade e Recursos Genéticos, InBIO, Laboratório Associado, Universidade do Porto, Vairão, Portugal

**Keywords:** European sardine, Sardina, genome, transcriptome, haplotype, single-nucleotide polymorphism

## Abstract

**Background:**

The European sardine (*Sardina pilchardus* Walbaum, 1792) is culturally and economically important throughout its distribution. Monitoring studies of sardine populations report an alarming decrease in stocks due to overfishing and environmental change, which has resulted in historically low captures along the Iberian Atlantic coast. Important biological and ecological features such as population diversity, structure, and migratory patterns can be addressed with the development and use of genomics resources.

**Findings:**

The genome of a single female individual was sequenced using Illumina HiSeq X Ten 10x Genomics linked reads, generating 113.8 gigabase pairs of data. Three draft genomes were assembled: 2 haploid genomes with a total size of 935 megabase pairs (N50 103 kilobase pairs) each, and a consensus genome of total size 950 megabase pairs (N50 97 kilobase pairs). The genome completeness assessment captured 84% of Actinopterygii Benchmarking Universal Single-Copy Orthologs. To obtain a more complete analysis, the transcriptomes of 11 tissues were sequenced to aid the functional annotation of the genome, resulting in 40,777 genes predicted. Variant calling on nearly half of the haplotype genome resulted in the identification of >2.3 million phased single-nucleotide polymorphisms with heterozygous loci.

**Conclusions:**

A draft genome was obtained, despite a high level of sequence repeats and heterozygosity, which are expected genome characteristics of a wild sardine. The reference sardine genome and respective variant data will be a cornerstone resource of ongoing population genomics studies to be integrated into future sardine stock assessment modelling to better manage this valuable resource.

## Data Description

### Background

**Figure 1:**
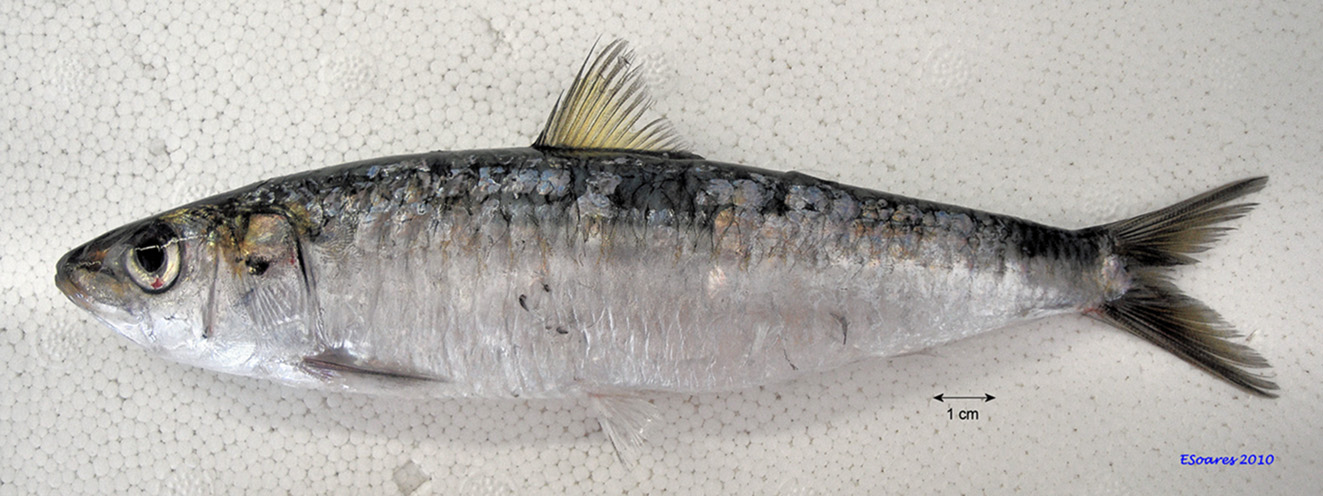
The European sardine, *Sardina pilchardus* (photo credit ©Eduardo Soares, IPMA)

The European sardine (*Sardina pilchardus* Walbaum, 1792) (National Center for Biotechnology Information [NCBI]: txid27697, Fishbase ID:1350) (Fig. 1) is a small pelagic fish occurring in temperate boundary currents of the Northeast Atlantic down to Cape Verde off the west coast of Africa, and throughout the Mediterranean to the Black Sea [1]. Two subspecies are generally recognized: *Sardina pilchardus pilchardus* occupies the northeastern Atlantic and the North Sea whereas *S. pilchardus sardina* occupies the Mediterranean and Black seas, and the North African coasts south to Cape Verde, with a contact zone near the Strait of Gibraltar [1, 2]. As with other members of the Clupeidae family (e.g., herring, *Clupea harengus*) and allis shad (*Alosa alosa*) [3], the sardine experiences strong population fluctuations in abundance, possibly reflecting environmental fluctuations, including climate change [4, 5].

The sardine is of major economic and social importance throughout its range, with a reported commercial catch for 2016 of 72,183 tonnes in European waters [6]. In Portugal, the sardine is an iconic and culturally revered fish and plays a central role in tourist events, such as summer festivals, throughout the country. However, recent stock assessment data strongly suggest that the Iberian sardine fisheries is under threat. A recent report by the International Council for the Exploration of the Sea [6] noted a sharp decrease in the Iberian Atlantic coast sardine stock and advised that catches in 2017 should be ≤23,000 tonnes. The sardine fishery biomass has experienced declining annual recruitment between 1978 and 2006, and more recently, it has fluctuated around historically low values, indicating a high risk of collapse of the Iberian Atlantic stocks [6].

A number of sardine populations have been identified by morphometric methods, including as many as 5 populations in the northeastern Atlantic (including the Azores), 2 off the Moroccan coast, and 1 in Senegalese waters [1, 7]. Each of these recognized sardine populations is subject to specific climatic and oceanic conditions, mainly during larval development, that directly influence the recruitment of the sardine fisheries [4, 8, 9]. However, because of phenotypic plasticity, morphological traits are strongly influenced by environmental conditions and the underlying genetics that defines those populations has proven elusive [10]. While the recognition of subspecies and localized populations might indicate significant genetic structure, the large population sizes and extensive migration of sardines are likely to increase gene flow and reduce population differences, suggesting, at its most extensive, a panmictic population with little genetic differentiation within the species’ range [11].

It is now well established that to fully understand the genetic basis of evolutionarily and ecologically significant traits, the gene and regulatory element composition of different individuals or populations needs to be assessed (see, e.g., [12, 13]). Therefore, we provide a European sardine draft genome, an essential tool to assess the genetic structure of the sardine population(s) and for genetic studies of the life history and ecological traits of this small pelagic fish, which will be instrumental for conservation and fisheries management.

### Genome sequencing

Sardines were caught during commercial fishing operations in the coastal waters off Olhão, Portugal, and maintained live at the experimental fish culture facilities (EPPO) of the Portuguese Institute for the Sea and Atmosphere (IPMA), Olhão, Portugal [14]. A single adult female was anesthetized with 2-phenoxyethanol (1:250 v/v), blood was collected in a heparinized syringe, and the fish killed by cervical section. Eleven tissues were dissected out—gill together with branchial arch, liver, spleen, ovary, midgut, white muscle, red muscle, kidney, head kidney, brain together with pituitary, and caudal fin (including skin, scales, bone, and cartilage)—into RNAlater (Sigma-Aldrich, Madrid, Spain) at room temperature followed by storage at −20°C. Fish maintenance and sample collection were carried out in accordance with the guidelines of the European Union Council (86/609/EU) and Portuguese legislation for the use of laboratory animals from the Veterinary Medicines Directorate (DGAV), the Portuguese competent authority for the protection of animals, Ministry of Agriculture, Rural Development and Fisheries, Portugal (permit 01 0238 of 19 April 2016).

Total RNA was extracted using a total RNA purification kit (Maxwell® 16 Total RNA Purification Kit, Promega) and digested twice with DNase (DNA-free kit, Ambion, UK). The total RNA samples were kept at −80°C until shipment to the RNA sequencing service provider Admera Health Co. (USA), which confirmed a RNA integrity number > 8 (Qubit Tapestation) upon arrival. The messenger RNA library preparation was performed with NEBNext^®^ Poly(A) mRNA Magnetic Isolation Module kit and NEBNext^®^ Ultra^™^ Directional RNA Library Prep kit for sequencing using Illumina HiSeq 4000 paired-end 150 base pair (bp) cycle to generate ∼596 million paired-end reads in total.

The genomic DNA (gDNA) was isolated from 20 µl of fresh blood using the DNeasy blood and tissue kit (Qiagen), followed by RNase treatment according to the manufacturer's protocol. The integrity of the gDNA was confirmed using pulsed-field gel electrophoresis and showed fragment sizes largely >50 kilobase pairs (kb). The gDNA was stored at −20°C before shipping to the service provider (Genome.one, Darlinghurst, Australia). Microfluidic partitioned gDNA libraries using the 10x Genomics Chromium System were made using 0.6 ng of gDNA input. Sequencing (150 bp paired-end cycle) was performed in a single lane of the Illumina HiSeq X Ten instrument (Illumina, San Diego, CA, USA). Chromium library size range (580–850 bp) was determined with LabChip GX Touch (PerkinElmer) and library yield (6.5–40 nM) by quantitative polymerase chain reaction.

### Genome size estimation

**Figure 2:**
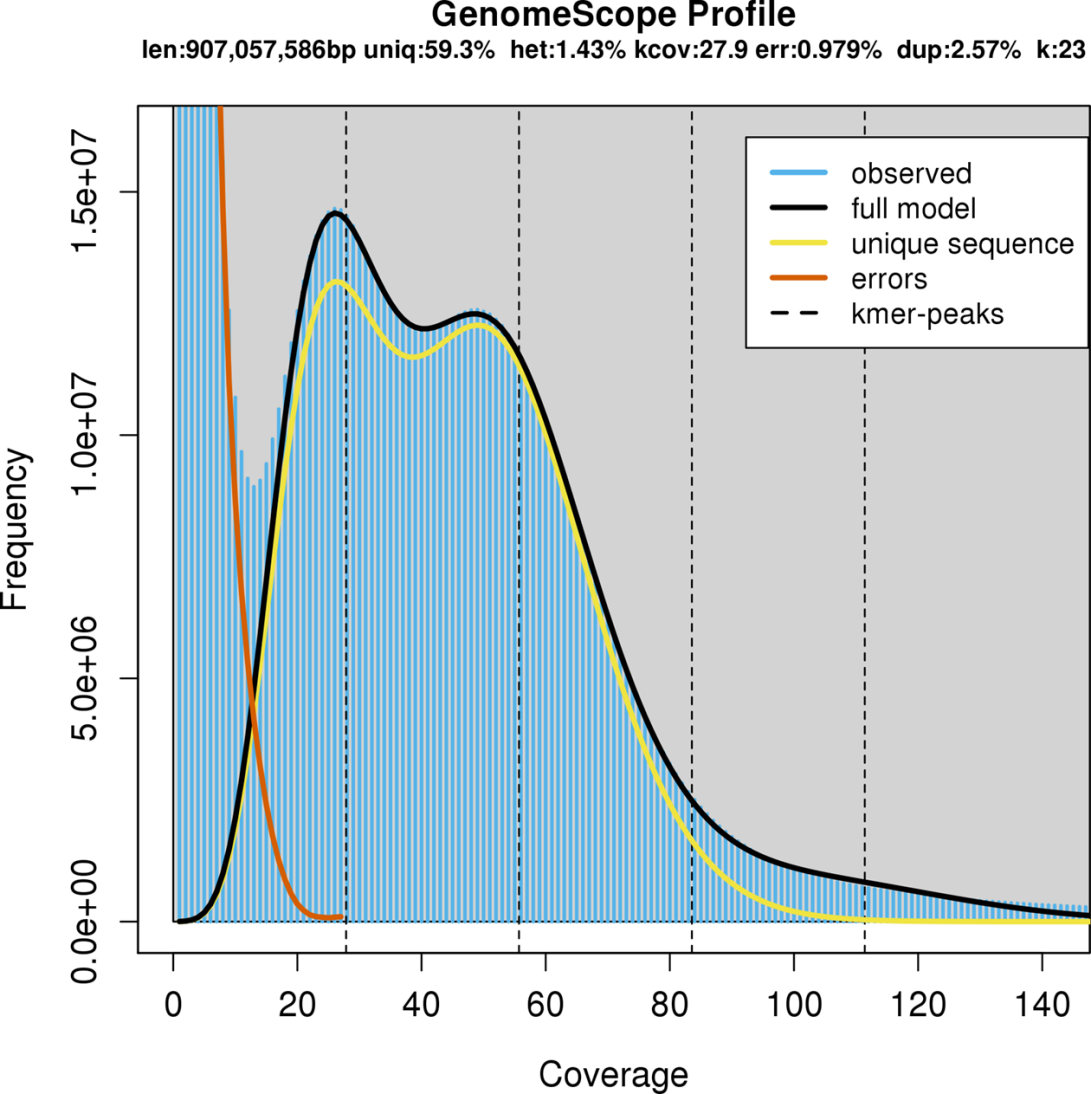
The histogram of the 23-mer depth distribution was plotted in GenomeScope [17] to estimate genome size (907 Mb), repeat content (40.7%), and heterozygosity level (1.43%). Two k-mer coverage peaks are observed at 28× and 50×.

A total of 759 million paired-end reads were generated, representing 113.8 gigabase pairs (Gb) of nucleotide sequences with 76.1% bases ≥ Q30. Raw reads were edited to trim 10x Genomics proprietary barcodes with a Python script “filter_10xReads.py” [15] prior to k-mer counting with Jellyfish v2.2.10 (Jellyfish, RRID:SCR_005491) [16]. A total of 670 million edited reads (90.5 Gb) were used to obtain the frequency distribution of 23-mers. The histogram of the k-mer counting distribution was plotted in GenomeScope v1.0.0 (GenomeScope, RRID:SCR_017014) [17] (Fig. 2) with maximum k-mer coverage of 10,000 for estimation of genome size, heterozygosity, and repeat content. The estimated sardine haploid genome size was 907 megabase pairs (Mb), with a repeat content of 40.7% and a heterozygosity level of 1.43% represented in the first peak of the distribution. These high levels of heterozygosity and repeat content indicated a troublesome genome characteristic for *de novo* assembly.

### *De novo* genome assembly

The *de novo* genome assembly was performed using the paired-end sequence reads from the partitioned library as input for the Supernova assembly algorithm v2.0.0 (7fba7b4) (Supernova assembler, RRID:SCR_016756) (10x Genomics, San Francisco, CA, USA) [18]. Two haplotype-resolved genomes, SP_haploid1 (European Nucleotide Archive [ENA] accession ID UOTT01000000) and SP_haploid2 (ENA accession ID UOTU01000000), were assembled with phased scaffolds using the Supernova “mkoutput pseudohap” option. For the assembly process the Supernova run parameters for maximum reads (–maxreads) and barcode fraction (–barfrac) were set for 650 million input reads and 80% of barcodes, respectively. Preliminary trials defined an optimal raw coverage of 78-fold, greater than the 56-fold suggested in the Supernova protocol; this reduced the problem (to some extent) of the complexity of the high repeat content (Table 1). A fraction of the 607.36 million read pairs were used after a quality control step embedded in the Supernova pipeline to remove reads that were not barcoded, not properly paired, or of low quality. Input reads had a 138.5-bp mean length after proprietary 10× barcode trimming and an N50 of 612 per barcode/DNA molecule (Table 1).

**Table 1:** Descriptive metrics, estimated by Supernova, of the input sequence data for the *de novo* genome assembly

Metric	Value
Number of paired reads used	607.36 million
Mean read length after trimming	138.50 bp
Median insert size	345 bp
Weighted mean DNA molecule size	46.41 kb
N50 reads per barcode	612
Raw coverage	78.35×
Effective read coverage	52.91×
Mean distance between heterozygous SNPs	197 bp

Further scaffolding and gap closure procedures were performed with Rails v1.2/Cobbler v0.3 pipeline script [19] to obtain the final consensus genome sequence named SP_G (ENA accession ID GCA_900 499 035.1) using the parameters anchoring sequence length (−*d* 100) and minimum sequence identity (*−i* 0.95). Three scaffolding and gap closure procedures were performed iteratively with 1 haplotype of the initial assembly as the assembly per se, and previous *de novo* assemblies from Supernova v1.2.2 (315 million/100% and 450 million/80% reads/barcodes). By closing several gaps within scaffolds and merging other scaffolds into longer and fewer scaffolds (117,259), this procedure resulted into a slightly longer genome size of 949.62 Mb, which slightly deflated the scaffold N50 length to 96.6 kb (Table 2). The assembly metrics of the 3 assemblies are described in Table 2, together with a recently published Illumina paired-end assembled sardine genome (UP_Spi) [20]. The total assembly size of our genome (SP_G) is 950 Mb and the UP_Spi is 641 Mb (Table 2). Because the SP_G and UP_Spi assembly sizes are of different orders of magnitude, in addition to N50 we present NG50 values [21] for an estimated genome size of 950 Mb (Table 2). In the SP_G assembly, 905 scaffolds (LG50) represents half of the estimated genome with an NG50 value of 96.6 kb, in comparison to LG50 of 15,422 and NG50 of 12.6 kb in the UP_Spi assembly. The ungapped length of the SP_G assembly is 828 Mb. The larger gaps of the SP_G assembly compared to the UP_Spi can be explained by the Supernova being able to estimate gap size based on the barcodes spanning the gaps, i.e., gaps have linkage evidence through the barcodes linking reads to DNA molecules and not solely gaps based on read pairs [22]. Such gaps are reflected in the large number of nucleotides per 100 kb in our assemblies (Table 2). The number of scaffolds in SP_G is 117,259 (largest, 6.843 Mb) and in UP_Spi is 44,627 (largest, 0.285 Mb).

**Table 2:** Descriptive metrics of sardine genome assemblies

Scaffolds	Spil_haploid1	Spil_haploid2	SP_G	UP_Spi
Largest	6.835 Mb	6.850 Mb	6.843 Mb	0.285 Mb
Number				
≥100 kb	874	872	890	309
≥10 kb	8,301	8,298	8,760	18,863
≥1 kb (total)	117,698	117,698	117,259	44,627
L50/N50				
≥100 kb	135/906.0 kb	134/925.2 kb	137/899.1 kb	130/122.5 kb
≥10 kb	242/572.7 kb	242/568.2 kb	254/552.2 kb	4,594/32.9 kb
≥1 kb (**total**)	859/102.9 kb	860/102.7 kb	903/96.6 kb	6,797/25.6 kb
LG50/NG50	935/87.7 kb	939/87.1 kb	905/96.6 kb	15,422/12.6 kb
Assembly size				
≥100 kb	469.371 Mb	468.838 Mb	473.550 Mb	39.274 Mb
≥10 kb	622.165 Mb	621.688 Mb	636.491 Mb	513.719 Mb
≥1 kb (**total**)	935.548 Mb	935.082 Mb	949.618 Mb	641.169 Mb
Guanine-cytosine content	43.9%	43.9%	43.9%	44.5%
Nucleotides per 100 kb	12,955	12,961	12,834	169

SP_haploid1/SP_haploid2: haploid genomes (UOTT01000000 and UOTU01000000). SP_G: consensus genome (NCBI representative genome assembly, GCA_900 499 035.1). UP_Spi: Illumina paired-end assembled genome from [20] (GCA_0 036 04335.1). Values for scaffolds ≥1, 10, and 100 kb are presented in separate rows.

The genome completeness assessment was estimated with Benchmarking Universal Single-copy Orthologs (BUSCO) v3.0.1 (BUSCO, RRID:SCR_015008) [23]. The genome was queried (options −m geno −sp zebrafish) against the “metazoa.odb9” lineage set containing 978 orthologs from 65 eukaryotic organisms to assess the coverage of core eukaryotic genes, and against the “actinopterygii.odb9” lineage set containing 4,584 orthologs from 20 different ray-finned fish species as the most taxon-specific lineage available for the sardine. Using the metazoan odb9 database, 95.4% of the genome had significant matches: 84.5% were complete genes (76.7% single-copy genes and 9.8% duplicates) and 8.9% were fragmented genes. By contrast, using the actinopterygii odb9 database, 84.2% (76.0% complete genes and 8.2% fragmented) had a match, with 69.3% of genes occurring as single copy and 6.7% as duplicates.

The EMBRIC configurator service [24] was used to create a fish-specific checklist (named finfish) for the submission of the sardine genome project to the ENA (ENA, RRID:SCR_006515) (project accession PRJEB27990).

### Repeat content

The SP_G consensus assembly was used as a reference genome to build a *de novo* repeat library running RepeatModeler v1.0.11 (RepeatModeler, RRID:SCR_015027) [25] with default parameters. The model obtained from RepeatModeler was used, together with Dfam_consensus database v20171107 [26] and RepBase RepeatMasker Edition library v20170127 [27], to identify repetitive elements and low-complexity sequences running RepeatMasker v4.0.7 (RepeatMasker, RRID:SCR_012954) [28]. The analysis carried out revealed that 23.33% of the assembled genome consists of repetitive elements.

### Genome annotation

The Maker v2.31.10 (MAKER, RRID:SCR_005309) [29] pipeline was used iteratively (5 times) to annotate the SP_G consensus genome. The annotations generated in each iteration were kept in the succeeding annotation steps and in the final General Feature Format (GFF) file. During the first Maker run the *de novo* transcriptome was mapped to the genome using blastn v2.7.1 (BLASTN, RRID:SCR_001598) [30] (est2genome parameter in Maker). Moreover, the repetitive elements found with RepeatMasker were used in the Maker pipeline. The initial gene models created by Maker were then used to train hidden Markov model (HMM)-based gene predictors. The preliminary GFF file generated by this first iteration run was used as input to train SNAP v2006-07-28 [31]. Using the scripts provided directly by Maker (maker2zff) and SNAP (fathom, forge, and hmm-assembler.pl) an HMM file was created and used as input for the next Maker iteration (snaphmm option in maker configuration file). For the next iteration, the gene-finding software Augustus v3.3 (Augustus, RRID:SCR_008417) [32] was self-trained running BUSCO with the specific parameter (–long), that turns on the Augustus optimization mode for self-training. The resulted predicted species model from Augustus was included in the pipeline in the third Maker run. For the fourth iteration, GeneMark-ES v4.32 (GeneMark, RRID:SCR_011930) [33], a self-training gene prediction software application, was executed and the resulting HMM file was integrated into the Maker pipeline. As further evidence for the annotation, in the last run of Maker, the genome was queried using blastx v2.7.1 (BLASTX, RRID:SCR_001653) (protein2genome parameter in Maker) against the deduced proteomes of herring (*C. harengus*, NCBI: txid7950, Fishbase ID:24) (GCF_000 966335.1), zebrafish (*Danio rerio*, NCBI: txid7955, Fishbase ID:4653) (GCF_0 00002035.6), blind cave fish (*Astyanax mexicanus*, NCBI: txid7994, Fishbase ID:2740) (GCF_000 372685.2), European sardine [20], and all proteins from teleost fishes in the UniProtKB/Swiss-Prot database (UniProtKB, RRID:SCR_004426) [34]. After the 5 Maker runs the selected 40,777 genes models are the *ab initio* predictions supported by the transcriptome and proteome evidence. Based on the transcriptomic evidence, 12,761 gene models were annotated with untranslated regions (UTR) features, more specifically 9,486 gene models with either 5′ or 3′ UTR and 3,275 gene models with both UTR features.

InterProScan v5.30 (InterProScan, RRID:SCR_005829) [35] and NCBI blastp v2.8.1 (BLASTP, RRID:SCR_001010) [30] were used to functionally annotate the 40,777 predicted protein coding genes. A total of 33,553 (82.3%) proteins were successfully annotated using blastp (e-value 1e−05) against the UniProtKB/Swiss-Prot database and another 5,228 were annotated using the NCBI non-redundant protein database. In addition to the above, 37,075 (90.9%) proteins were successfully annotated using InterProScan with all the InterPro v72.0 (InterPro, RRID:SCR_006695) [36] databases: CATH-Gene3D (Gene3D, RRID:SCR_007672), Hamap (HAMAP, RRID:SCR_007701), PANTHER (PANTHER, RRID:SCR_004869), Pfam (Pfam, RRID:SCR_004726), PIRSF (PIRSF, RRID:SCR_003352), PRINTS (PRINTS, RRID:SCR_003412), ProDom (ProDom, RRID:SCR_006969), ProSite Patterns (PROSITE, RRID:SCR_003457), ProSite Profiles, SFLD (Structure-function linkage database, RRID:SCR_001375), SMART (SMART, RRID:SCR_005026), SUPERFAMILY (SUPERFAMILY, RRID:SCR_007952), and TIGRFAM (JCVI TIGRFAMS, RRID:SCR_005493). In total, 38,880 (95.3%) of the predicted proteins received a functional annotation. The annotated genome assembly is published [37] in the wiki-style annotation portal ORCAE [38].

**Figure 3:**
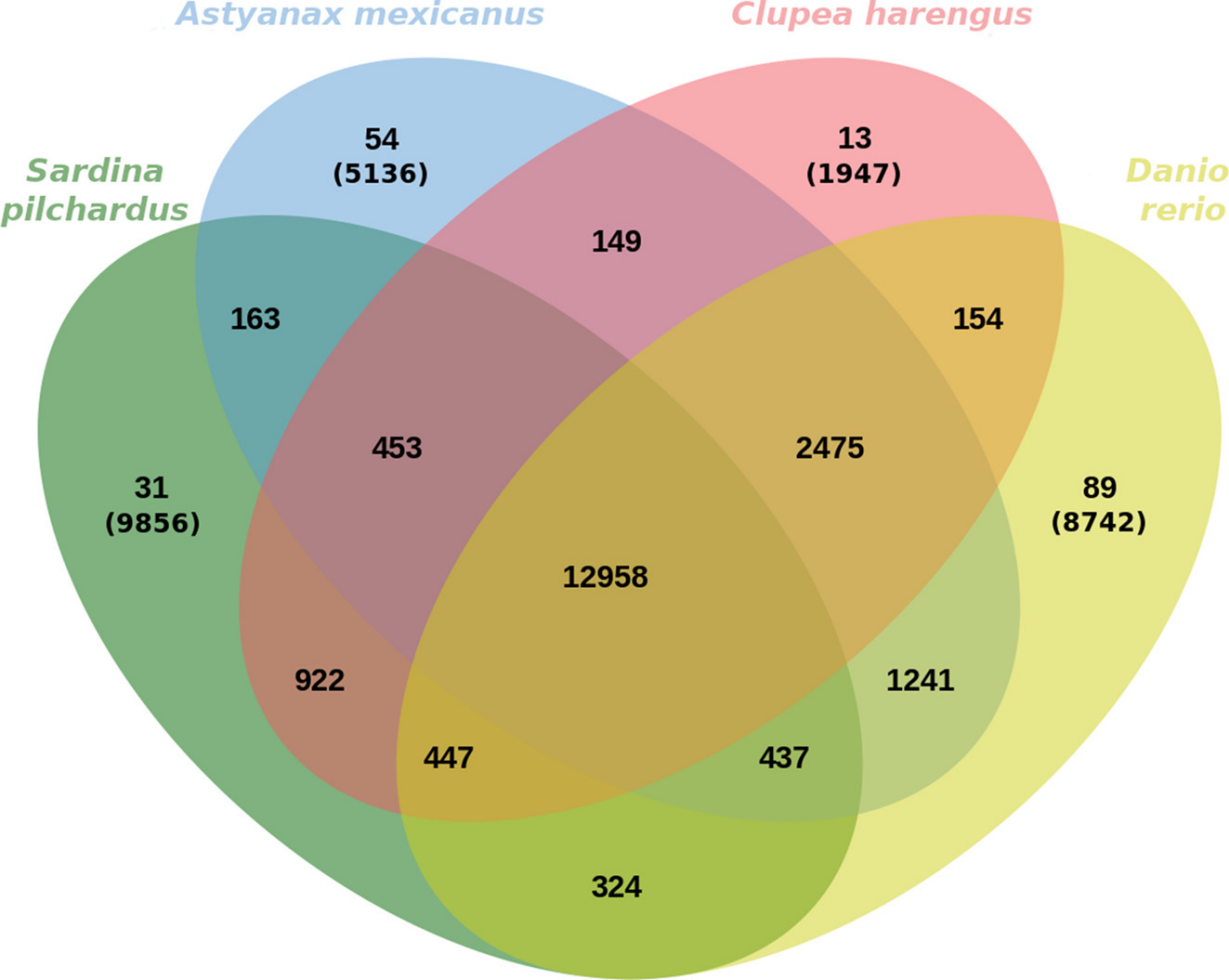
Venn diagram representing paralogous and orthologous groups between sardine (*S. pilchardus*), blind cave fish (*A. mexicanus*), zebrafish (*D. rerio*), and herring (*C. harengus*) obtained with OrthoFinder and plotted with Jvenn [40]. Orthogroups of singleton genes are showed in parentheses.

OrthoFinder v2.2.7 [39] was used to identify paralogy and orthology in our Swiss-prot annotated deduced proteome and in the deduced proteomes from herring, blind cave fish, and zebrafish. The resulting orthogroups were plotted using jvenn (jVenn, RRID:SCR_016343) [40] (Fig. 3), where paralagous (≥2 genes) and singletons were identified within species-specific orthogroups. The deduced sardine proteome has 3,413 paralogous groups containing 11,406 genes, of which 31 are sardine-specific orthogroups. The amount of sardine singletons (9,856) can be partially due to fragmented predicted genes but can also reflect some evolutionary divergence, which requires further study to understand the biological relevance. In total, 25,560 orthogroups containing at least a single protein were identified in sardine, of which 12,958 ortholgroups are common to all 4 fish species. Within the Clupeidae, the sardine and the herring share 14,780 orthogroups with 922 family-specific orthogroups.

### Variant calling between phased alleles

FASTQ files were processed using the 10x Genomics LongRanger v2.2.2 pipeline [41] with a maximum input limit of 1,000 scaffolds, defined as reference genome, and representing approximately half of the genome size (488.5 Mb). The LongRanger pipeline was run with default settings, with the exception of vcmode to define the Genome Analysis Toolkit (GATK) v4.0.3.0 (GATK, RRID:SCR_001876) [42] as the variant caller and the somatic parameters. The longest phase block was 2.86 Mb and the N50 phase block was 0.476 Mb.

Single-nucleotide polymorphisms (SNPs) were furthered filtered to obtain only phased and heterozygous SNPs between the 2 alleles with a coverage >10-fold using VCFtools v0.1.16 (VCFtools, RRID:SCR_001235). A VCF file was obtained containing 2,369,617 filtered SNPs (Additional file 1), resulting in a mean distance between heterozygous phased SNPs of 206 bp. Similar results were obtained in the Supernova input report estimation (Table 1) of mean distance between heterozygous SNPs in the whole genome of 197 bp. This high SNP heterozygosity (1/206), observed solely in the comparison of the phased alleles, is higher than the average nucleotide diversity of the previously reported marine fish of wild populations: 1/390 in yellow drum [43], 1/309 in herring [44], 1/435 in coelacanth [45], 1/500 in cod [46], and 1/700 in stickleback [47].

### *De novo* transcriptome assembly

The 596 million paired-end raw transcriptomic reads were edited for contamination (e.g., adapters) using TrimGalore v0.4.5 wrapper tool (TrimGalore, RRID:SCR_016946) [15], low-quality base trimming with Cutadapt v1.15 (cutadapt, RRID:SCR_011841) [48], and the output overall quality reports of the edited reads with FastQC v0.11.5 (FastQC, RRID:SCR_014583) [49].

The 553 million edited paired-end reads were *de novo* assembled as a multi-tissue assembly using Trinity v2.5.1 (Trinity, RRID:SCR_013048) [50] with a minimum contig length of 200 bp, 50× coverage read depth normalization, and replicative form strand-specific read orientation. The same parameters were used for each of the 11 tissue-specific *de novo* assemblies. The genome and transcriptome assemblies were conducted on the Portuguese National Distributed Computing Infrastructure [49].

The 12 *de novo* transcriptome assemblies (Table 3) were each quality assessed using TransRate v1.0.3 [51] with read evidence for assembly optimization, by specifying the contigs fasta file and respective left and right edited reads to be mapped. The multi-tissue assembly with all reads resulted in an assembled transcriptome of 170,478 transcript contigs following the TransRate step. Functional annotation was performed using the Trinotate v3.1.1 pipeline [24] and integrated into a SQLite database. All annotations were based on the best deduced open reading frame (ORF) obtained with the Transdecoder v1.03 [51]. Of the 170,478 transcript contigs, 27,078 (16%) were inferred to ORF protein sequences. Query of the UniProtKB/Swiss-Prot (e-value cutoff of 1e−5) database via blastx v2.7.1 of total contigs resulted in 43,458 (26%) annotated transcripts. The ORFs were queried against UniProtKB/Swiss-Prot (e-value cutoff of 1e−5) via blastp v2.7.1 and PFAM using hmmscan (HMMER v3.1b2) (Hmmer, RRID:SCR_005305) [52], resulting in 19,705 (73% of ORF) and 16,538 (61% of ORF) UniProtKB/Swiss-Prot and PFAM annotated contigs, respectively. The full annotation report with further functional annotation, such as signal peptides, transmembrane regions, eggnog, Kyoto Encyclopedia of Genes and Genomes (KEGG) (KEGG, RRID:SCR_012773), and Gene Ontology annotation (Gene Ontology, RRID:SCR_002811) is listed in tabular format in Additional file 2.

**Table 3:** Summary statistics of transcriptome data for the 11 tissues

Tissue	Paired raw reads	Contigs	CDS deduced (%)	SwissProt annotated (%)	Accession No.
Gill/branchial arch	29,783,994	62,526	29.3	38.6	ERS2629269
Liver	33,479,471	53,104	29.7	40.1	ERS2629273
Spleen	25,634,530	66,419	31.6	40.4	ERS2629276
Ovary	22 241,327	42,521	38.1	42.5	ERS2629270
Midgut	28,016,117	75,782	31.0	39.5	ERS2629274
White muscle	24,409,160	49,266	35.4	44.8	ERS2629277
Red muscle	30,653,774	55,873	30.3	42.1	ERS2629275
Kidney	27,861,879	59,495	30.8	37.3	ERS2629272
Head kidney	25,280,960	65,888	32.2	38.4	ERS2629271
Brain/pituitary	24,467,352	75,620	24.5	37.1	ERS2629267
Caudal fin (skin/cartilage/bone)	26,342,097	64,832	23.9	38.0	ERS2629268
All tissues	298,170,661	170,478	15.9	25.5	ERS2629362

### Ray-finned fish phylogeny

We conducted a phylogenetic analysis of ray-finned fish (Actinopterygii) taxa based on 17 fish species. The sardine protein dataset used in the phylogenetic analysis was obtained by querying the deduced proteins from our sardine genome against the 1-to-1 orthologous cluster dataset (106 proteins from 17 species) obtained from Machado et al. [20].

For the query, gene models were constructed for each protein with hmmbuild (HMMER v3.1b2) [53] using default options and the orthologous genes from the deduced sardine proteome were searched using hmmsearch (HMMER) with an e-value cuttoff of 10e−3. The best protein hits, as indicated by the bitscores, were aligned to the original protein sequence alignments using hmmalign (HMMER) with default options. Gapped and poorly aligned sites were identified by Gblocks v0.91b (Gblocks, RRID:SCR_015945) [54] using default options and removed using p4 v1.3.0 [55]. Protein alignment statistics were calculated, and the proteins concatenated into a single alignment using novel scripts in p4. Of the 106 fish protein alignments, 97 contained sites that were considered correctly aligned by the Gblocks analysis; statistics for these alignments are presented in Table S1 (Additional file 3). The concatenated sequence alignment of the 97 proteins contained 14,515 sites without gaps of which 7,391 were constant, 7,123 variable, and 3,879 parsimony informative.

The best-fitting empirical protein model of the concatenated data was evaluated using ModelFinder [56] in IQ-TREE v1.6.7.1 [57]. The best-fitting empirical substitution model was estimated to be the JTT model [58] with a discrete gamma-distribution of among-site rate variation (4 categories) and empirical composition frequencies (typical notation: JTT+Г_4_+F).

**Figure 4:**
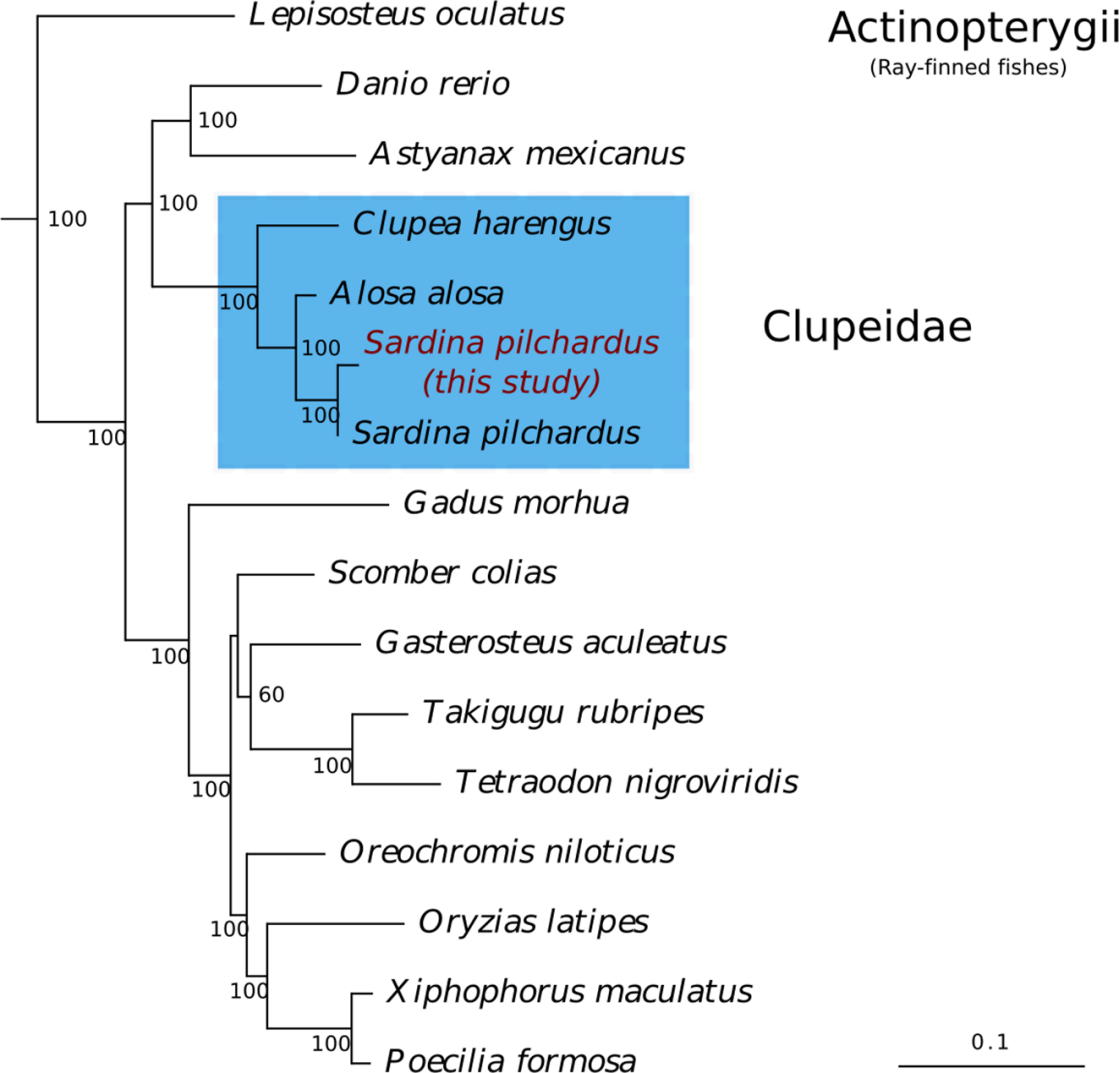
Optimal maximum likelihood tree (−ln likelihood: 146,565.6438) under a best-fitting JTT+Г_4_+F substitution model of 97 concatenated proteins. Maximum likelihood bootstrap support values are given below or to the right of nodes. Scale bar represents mean numbers of substitutions per site. The Actinopterygii ingroup was rooted to 2 outgroup taxa, namely, *Petromyzon marinus* (lamprey) and *Latimeria chalumnae* (coelacanth) (not shown).

Optimal maximum likelihood tree searches (100 replicates) and bootstrap analyses (300 replicates) were conducted using RAxML v8.2.12 (RAxML, RRID:SCR_006086) [59] with the best-fitting model. The optimal maximum likelihood tree (−ln likelihood: 146,565.6438) is presented in Fig. 4 with bootstrap support values given at nodes and is rooted to the outgroups *Petromyzon marinus* (lamprey) and *Latimeria chalumnae* (coelacanth).

## Conclusion

Despite the sardine genome having a high level of repeats and heterozygosity, factors that pose a challenge to *de novo* genome assembly, a more than adequate draft genome was obtained with the 10x Genomics linked-reads (Chromium) technology. The Chromium technology's ability to tag and cluster the reads to individual DNA molecules has proven advantages for scaffolding, just like long-read technologies such as Nanopore and Pacific Biosciences, but with high coverage and low error rates. The advantage of linked reads for *de novo* genomic assemblies is evident in comparison with typical short-read data, especially in the case of wild species with highly heterozygous genomes, where the latter often result in many uncaptured genomic regions and with a lower scaffolding yield due to repeated content.

The high degree of heterozygosity identified here in the sardine genome illustrates future problems for monitoring sardine populations using low-resolution genetic data. However, the phased SNPs obtained in this study can be used to initiate the development of a SNP genetic panel for population monitoring, with SNPs representative of haplotype blocks, allowing insights into the patterns of linkage disequilibrium and the structure of haplotype blocks across populations.

The genomic and transcriptomic resources reported here are important tools for future studies to elucidate sardine response at the levels of physiology, population genetics, and ecology of the causal factors responsible for the recruitment and collapse of the sardine stock on the Iberian Atlantic coast. Besides the commercial interest, the sardine plays a crucial role at a key trophic level by bridging energy from the primary producers to the top predators in the marine ecosystem. Therefore, disruption of the sardine population equilibrium is likely to reverberate throughout the food chain via a trophic cascade. Consequently, these genomic and genetic resources are the prerequisites needed to develop tools to monitor the population status of the sardine and thereby provide an important bio-monitoring system for the health of the marine environment.

## Availability of supporting data and materials

Raw data, assembled transcriptomes, and assembled genomes are available at the European Bioinformatics Institute ENA archive with the project accession PRJEB27990. The annotated genome assembly is published in the wiki-style annotation portal ORCAE [37]. Supporting data and materials are available in the *GigaScience* GigaDB database [60].

## Additional files

**Additional file 1**. Heterozygous SNPs identified in the phased haploid blocks listed in a VCF file format.

**Additional file 2**. Annotation of all tissues transcriptome assembly in a tabular format.

**Additional file 3**. Sequence alignment statistics of the 97 proteins concatenated for the phylogenetics analyses.

GIGA-D-18-00377_Original_Submission.pdf

GIGA-D-18-00377_Revision_1.pdf

GIGA-D-18-00377_Revision_2.pdf

Response_to_Reviewer_Comments_Original_Submission.pdf

Response_to_Reviewer_Comments_Revision_1.pdf

Reviewer_1_Report_Original_Submission -- Andrew Thompson 10/29/2018 Reviewed

Reviewer_1_Report_Revision_1 -- Andrew Thompson 3/29/2019 Reviewed

Reviewer_2_Report_Original_Submission -- Shigehiro Kuraku 11/4/2018 Reviewed

Reviewer_2_Report_Revision_1 -- Shigehiro Kuraku 4/3/2019 Reviewed

Supplemental Files

## Abbreviations

bp: base pair; BUSCO: Benchmarking Universal Single-copy Orthologs; DGAV: Veterinary Medicines Directorate; ENA: European Nucleotide Archive; EPPO: experimental fish culture facilities; Gb: gigabase pairs; gDNA: genomic DNA; GFF: General Feature Format; HMM: hidden Markov model; IPMA: Portuguese Institute for the Sea and Atmosphere; kb: kilobase pairs; KEGG: Kyoto Encyclopedia of Genes and Genomes; Mb: megabase pairs; NCBI: National Center for Biotechnology Information; ORF: open reading frame; SNP: single-nucleotide polymorphism; UTR: untranslated region.

## Competing interests

The authors declare that they have no competing interests.

## Funding

This research was supported by Portuguese national funds from FCT—Foundation for Science and Technology through project UID/Multi/04326/2016 and by FCT and the European Regional Development Fund (FEDER) under projects 22153-01/SAICT/2016 (to the National Infrastruture of Distributed Computing of Portugal), ALG-01-0145-FEDER-022121 and ALG-01-0145-FEDER-022231; and co-funds from MAR2020 operational programme of the European Maritime and Fisheries Fund (project SARDINOMICS MAR-01.04.02-FEAMP-0024). The EMBRIC configurator service received funding from the European Union's Horizon 2020 research and innovation programme under grant agreement No. 654008.

## Authors' contributions

Writing original draft: B.L., G.D.M., C.J.C.; investigation: B.L., G.D.M., C.G.; review and editing: A.V., S.J.S., A.M.S., A.V.M.C.; conceptualization: B.L., G.D.M., C.J.C., A.V., S.J.S., A.M.S., A.V.M.C.
