# Supplementary material for: A haplotype-resolved draft genome of the European sardine (Sardina pilchardus)
Source: Gigascience. 2019 May 21;8(5):giz059. doi: 10.1093/gigascience/giz059 (PMC6528745; doi:10.1093/gigascience/giz059)
Supplement: GIGA-D-18-00377_Original_Submission.pdf [file giz059_giga-d-18-00377_original_submission.pdf]

GigaScience

A haplotype-resolved draft genome of the European sardine (Sardina Pilchardus)

--Manuscript Draft--

**Manuscript Number:**	GIGA-D-18-00377
**Full Title:**	A haplotype-resolved draft genome of the European sardine (Sardina Pilchardus)
**Article Type:**	Data Note
**Funding Information:**	H2020 Research Infrastructures (654008) — Not applicable<br>Fundação para a Ciência e a Tecnologia (UID/Multi/04326/2013) — Not applicable<br>Fundação para a Ciência e a Tecnologia (22153-01/SAICT/2016) — Not applicable

Abstract:

Background

The European sardine (Sardina pilchardus Walbaum, 1792) has a high cultural and economic importance throughout its distribution. Monitoring studies of the sardine populations report an alarming decrease in stocks due to overfishing and environmental change. There is an urgent need to better understand the causal factors of this continuous decrease in the sardine stock, which has recorded a low historical level in the Iberian Atlantic coast. Important biological and ecological features such as levels of population diversity, structure, and migratory patterns can be addressed with the development and use of genomics resources.

Findings

The sardine genome of a single female individual was sequenced using Illumina HiSeq X Ten 10X Genomics linked-reads generating 113.8Gb of sequencing data. Two haploid and a consensus draft genomes were assembled, with a total size of 935 Mbp (N50 103 Kb) and 950Mbp (N50 97 Kb), respectively. The genome completeness assessment captured 84% of Actinopterygii Benchmarking Universal Single-Copy Orthologs. To obtain a more complete analysis the transcriptomes of eleven tissues were sequenced and used to aid the functional annotation of the genome resulting in 29,408 genes predicted. Variant calling on nearly half of the haplotype genome resulted in the identification of more than 2.3 million phased SNPs with heterozygous loci.

Conclusions

The sardine genome is a cornerstone for future population genomics studies, the results of which may be integrated into future sardine stock modelling to better manage this valuable resource.

**Corresponding Author:**	Adelino V. M. Canário<br><br>PORTUGAL
**Corresponding Author Secondary Information:**	
**Corresponding Author's Institution:**	
**Corresponding Author's Secondary Institution:**	
**First Author:**	Bruno Louro, PhD
**First Author Secondary Information:**	
**Order of Authors:**	Bruno Louro, PhD
	Gianluca De Moro, PhD
	Carlos Garcia

Powered by Editorial Manager® and ProduXion Manager® from Aries Systems Corporation

	Cymon J. Cox, PhD
	Ana Veríssimo
	Stephen J. Sabatino
	António M. Santos
	Adelino V. M. Canário, PhD
**Order of Authors Secondary Information:**	
**Additional Information:**	
**Question**	**Response**
Are you submitting this manuscript to a special series or article collection?	No
**Experimental design and statistics**<br><br>Full details of the experimental design and statistical methods used should be given in the Methods section, as detailed in our Minimum Standards Reporting Checklist. Information essential to interpreting the data presented should be made available in the figure legends.<br><br>Have you included all the information requested in your manuscript?	Yes
**Resources**<br><br>A description of all resources used, including antibodies, cell lines, animals and software tools, with enough information to allow them to be uniquely identified, should be included in the Methods section. Authors are strongly encouraged to cite Research Resource Identifiers (RRIDs) for antibodies, model organisms and tools, where possible.<br><br>Have you included the information requested as detailed in our Minimum Standards Reporting Checklist?	Yes
**Availability of data and materials**<br><br>All datasets and code on which the conclusions of the paper rely must be	Yes

either included in your submission or deposited in publicly available repositories (where available and ethically appropriate), referencing such data using a unique identifier in the references and in the "Availability of Data and Materials" section of your manuscript.<br><br>Have you have met the above requirement as detailed in our Minimum Standards Reporting Checklist?	

Powered by Editorial Manager® and ProduXion Manager® from Aries Systems Corporation

Manuscript
Click here to download Manuscript Louro_et_al_manuscript.docx

A haplotype-resolved draft genome of the European sardine (*Sardina Pilchardus*)

Bruno Louro[1]*; Gianluca De Moro[1]*; Carlos Garcia[1]; Cymon J. Cox[1]; Ana Veríssimo[2]; Stephen J. Sabatino[2]; António M. Santos[2]; Adelino V. M. Canário[1&]

1 CCMAR Centre of Marine Sciences, University of Algarve, Campus de Gambelas, 8005-139 Faro, Portugal.

2 CIBIO, Centro de Investigação em Biodiversidade e Recursos Genéticos, InBIO, Laboratório Associado, Universidade do Porto, Vairão, Portugal

* authors contributed equally

[&] Corresponding author: Adelino V. M. Canário, e-mail: acanario@ualg.pt

Abstract

Background: The European sardine (*Sardina pilchardus* Walbaum, 1792) has a high cultural and economic importance throughout its distribution. Monitoring studies of the sardine populations report an alarming decrease in stocks due to overfishing and environmental change. There is an urgent need to better understand the causal factors of this continuous decrease in the sardine stock, which has recorded a low historical level in the Iberian Atlantic coast. Important biological and ecological features such as levels of population diversity, structure, and migratory patterns can be addressed with the development and use of genomics resources. **Findings:** The sardine genome of a single female individual was sequenced using Illumina HiSeq X Ten 10X Genomics linked-reads generating 113.8Gb of sequencing data. Two haploid and a consensus

draft genomes were assembled, with a total size of 935 Mbp (N50 103 Kb) and 950Mbp (N50 97 Kb), respectively. The genome completeness assessment captured 84% of Actinopterygii Benchmarking Universal Single-Copy Orthologs. To obtain a more complete analysis the transcriptomes of eleven tissues were sequenced and used to aid the functional annotation of the genome resulting in 29,408 genes predicted. Variant calling on nearly half of the haplotype genome resulted in the identification of more than 2.3 million phased SNPs with heterozygous loci. **Conclusions:** The sardine genome is a cornerstone for future population genomics studies, the results of which may be integrated into future sardine stock modelling to better manage this valuable resource.

Keywords: European sardine; Sardina; genome; transcriptome; haplotype; SNP

Data description

Background

The European sardine (*Sardina pilchardus* Walbaum, 1792) (Figure 1) is a small pelagic fish occurring in temperate boundary currents of the Northeast Atlantic down to Cape Verde off the west coast of Africa, and throughout the Mediterranean to the Black Sea. Two subspecies are generally recognised: *Sardina pilchardus pilchardus* occupies the north-eastern Atlantic and the North Sea whereas *S. pilchardus sardina* occupies the Mediterranean and Black seas, and the North African coasts south to Cape Verde, with a contact zone near the Strait of Gibraltar [1, 2]. As with other members of the Clupeidae family (e.g. herring, *Clupea harengus*, Allis shad, *Alosa*

alosa) [3], the sardine experiences strong population fluctuations, possibly reflecting environmental fluctuations, including climate change [4, 5].

The sardine is of major economic and social importance throughout its range with a reported commercial catch for 2016 of 72,183 tonnes in European waters. Indeed, in a country such as Portugal the sardine is an iconic and culturally revered fish which plays a central role in touristic events such as summer festivals throughout the country. However, recent fisheries data strongly suggests the Portuguese sardine fisheries are under threat. A recent report the International Council for the Exploration of the Sea [6] noted sharp decreases in the Iberian Atlantic coast sardine stock that resulted in ICES advice that catches in 2017 should be no more than 23,000 tonnes. The sardine fishery biomass has suffered from a declining trend of annual recruitment between 1978 and 2006 and more recently it fluctuates around historically low values, with a high risk of collapse of the Iberian Atlantic stocks [6].

A number of sardine stocks have been identified by morphometric methods, including as many as five stocks in the north-eastern Atlantic (including the Azores), two off the Moroccan coast, and one in Senegalese waters [1, 7]. Each of these recognized sardine stocks is subjected to specific climatic and oceanic conditions, mainly during larval development and recruitment, which directly influence the recruitment of the sardine fisheries in the short term [4, 8, 9]. However, because of phenotypic plasticity, morphological traits are strongly influenced by environmental conditions and the underlying genetics that define those stocks has proven elusive [10]. While the recognition of subspecies and localised stocks might indicate significant genetic structuring of the population, the large population sizes and extensive migration of sardines are likely to increase gene flow and reduce differences among stocks,

suggesting, at its most extensive, a panmictic population with little genetic differentiation within the species' range [11].

It is now generally well established that to fully understand the genetic basis of evolutionarily and ecologically significant traits, the gene and regulatory element composition at the genomic level needs to be assessed [see e.g., 12, 13]. Therefore, here we provide a European sardine draft genome to serve as a tool for conservation and fisheries management, providing the essential context to assess the genetic structure of the sardine population(s) and for baseline studies of the genetic basis of the life-history and ecological traits of this small pelagic.

Genome sequencing

Sardines were caught during commercial operations in the coastal waters off Olhão, Portugal, and maintained live at the experimental fish culture facilities (EPPO) of the Portuguese Institute for the Sea and Atmosphere (IPMA) in Olhão, Portugal [14]. A single adult female was anesthetised with 2-phenoxyethanol (1:250 v/v), blood sampled with a heparinized syringe, and euthanized by cervical section. Eleven tissues were dissected out - gill plus branchial arch, liver, spleen, female gonad, midgut, white muscle, red muscle, kidney, head kidney, brain plus pituitary and caudal fin (including skin, scales, bone and cartilage) – into RNA*later* (Sigma-Aldrich, USA) at room temperature followed by storage at −20 °C. The tissue sampling was carried out in accordance with the Guidelines of the European Union Council (86/609/EU) and Portuguese legislation for the use of laboratory animals, under licence (Permit number 010238 from 19/04/2016) from the Veterinary Medicines Directorate (DGAV), the Portuguese competent authority for the protection of animals, Ministry of Agriculture, Rural Development and Fisheries, Portugal.

Total RNA was extracted using a total RNA purification kit (Maxwell® 16 Total RNA Purification Kit, Promega) and digested twice with DNase (DNA-free kit, Ambion, UK). The total RNA samples where kept at -80ºC until shipment to the RNAseq service provider Admera Health Co. (USA) which confirmed a RIN above 8 (Qubit Tapestation) upon arrival. The mRNA library preparation was performed with NEBNext® Poly(A) mRNA Magnetic Isolation Module kit and NEBNext® Ultra™ Directional RNA Library Prep kit for posterior sequencing using Illumina HiSeq 4000 paired-end 150 bp cycle to generate about 596 million paired-end reads in total.

The genomic DNA (gDNA) was isolated from 20 µl of fresh blood using the DNeasy blood and tissue kit (Quiagen), followed by RNase treatment according to the manufacturer's protocol. The integrity of the gDNA was confirmed using pulsed-field gel electrophoresis and showed a molecular weight largely above 50 kbp. The gDNA was stored at −20 °C before shipping to the service provider (genome.one, Darlinghurst, Australia). Microfluidic partitioned gDNA libraries using the 10x Genomics Chromium System were made using 0.6 ng of gDNA input. Sequencing (150bp paired-end cycle) was performed in a single lane of the Illumina HiSeq X Ten instrument (Illumina, San Diego, CA, USA). Chromium library size range (580-850 bp) was determined with LabChip GX Touch (PerkinElmer) and library yield (6.5-40 ɲM) by quantitative polymerase chain reaction.

Genome size estimation

Seven hundred and fifty nine million paired-end reads were generated representing 113.8 Gb nucleotide sequences with 76.1% bases >= Q30. Raw reads were edited to trim 10X Genomics proprietary barcodes with a python script "filter_10xReads.py" [15] prior to kmer counting with Jellyfish v2.2.10 [16]. Six hundred and seventy million

edited reads (90.5 Gb) were used to obtain the frequency distribution of 23-mers. The histogram of the kmer counting distribution was plotted in GenomeScope [17] (Figure 2) with maximum kmer coverage of 10,000 for estimation of genome size, heterozygosity and repeat content. The estimated sardine haploid genome size was 907Mbp with a repeat content of 40.7% and a heterozygosity level of 1.43% represented in the first peak of the distribution. These high levels of heterozygosity and repeat content indicated a troublesome genome characteristic of *de novo* assembly.

De novo genome assembly

The de-novo genome assembly was done using the paired-end sequence reads from the partitioned library as input for the Supernova assembly algorithm (version 2.0.0(7fba7b4), 10x Genomics, San Francisco, CA, USA) [18] to output two haplotype-resolved genomes with phased scaffolds using the Supernova mkoutput pseudohap option. For the assembly process the Supernova run parameters for maximum reads (--maxreads) and barcode fraction (--barfrac) were set for 650M input reads and 80% of barcodes, respectively. Preliminary trials defined an optimal raw coverage of 78-fold, above the 56-fold suggested in the Supernova protocol; this allowed tackling (to some extent) the complexity of the high repeat content nature of the genome in the assembly (Table 1). Of the defined raw reads maximum input, a fraction of 607.36 million read pairs were used after a quality editing step embedded in the Supernova pipeline to remove reads that were not barcoded, not properly paired or low-quality reads. Input reads had a 138.5 bp mean length after proprietary 10X barcode trimming and a N50 of 612 per barcode/DNA molecule (Table 1).

Further scaffolding and gap closure procedures were performed with Rails v1.2/Cobbler v0.3 pipeline script [19] to obtain the final consensus genome sequence using the parameters anchoring sequence length (*-d* 100) and minimum sequence identity (*-i* 0.95). Three scaffolding and gap procedures were performed iteratively with one haplotype of the initial assembly as the assembly *per se*, and previous *de novo* assemblies from Supernova (version 1.2.2), (315M/100% and 450M/80% reads/barcodes). By closing several gaps within scaffolds and merging other scaffolds into longer and fewer scaffolds (117,259), this procedure resulted into a slightly longer genome size of 949.62 Mb, which deflated slightly the scaffold N50 length to 96.6 Kb (Table 2).

The genome completeness assessment was estimated with Busco v3.0.1 [20]. About 83.7% and 91.8% of the genome had significant matches against the actinopterygii and eukaryota odb9 databases, respectively. The actinopterygii.odb9 contains 4584 orthologs from 20 different species, and the eukaryota.odb9 contains 303 orthologs from sixty-five eukaryotic organisms.

The EMBRIC configurator service [21] was used to create a finfish checklist for the submission of the sardine genome project to the European Nucleotide Archive (ENA) (project accession PRJEB27990).

Repeat Content

The Spil assembly was used as a reference genome to build a *de novo* repeat library running RepeatModeler v1.0.11 [22] with default parameters. The model obtained from RepeatModeler was used, together with Dfam_consensus database v. 20171107 [23] and RepBase RepeatMasker Edition library v. 20170127 [24] to identify repetitive elements and low complexity sequences running RepeatMasker (v. 4.0.7) [25]. The

analysis carried out revealed that 23.33% of the assembled genome harbours at least one repeat.

Genome annotation

The RNA-seq assembly, repetitive elements, protein homology and *ab initio* gene prediction were used in a custom annotation pipeline based on multiple runs of Maker v. 2.31.10 [26]. The final high quality gene models were obtained using a *de novo* trained set from SNAP v. 2006-07-28 [27], Augustus v. 3.3 [28] and the self-training software GeneMark v. 4.32 [29]. The trained file for SNAP was generated using the output of the first run of Maker and the Augustus run was trained using the specific option in Busco v3.0.1 [20]. The pipeline identified 29,408 genes.

Interproscan v. 5.30 [30] and NCBI blastp v. 2.6 [31] were used to functionally annotate the 30,169 predicted protein coding genes. Thirteen thousand five hundred and fifty nine (44.9%) proteins were successfully annotated using blastp (e-value 1e-05) against the SwissProt database [32] and another 2,499 were annotated using the NCBI non-redundant protein database (NR). In addition to the above, 17,132 (56.8%) proteins were successfully annotated running interproscan with all the interpro v. 69.0 [33] databases (CDD, CATH-Gene3D, Hamap, PANTHER, Pfam, PIRSF, PRINTS, ProDom, ProSite Patterns, ProSite Profiles, SFLD, SMART, SUPERFAMILY, TIGRFAM). In total, 17,199 (65%) of the predicted proteins received a functional annotation. The annotated genome assembly is published [34] in the wiki-style annotation portal ORCAE [35] .

Variant calling between phased alleles

FASTQ files were processed using 10x Genomics LongRanger v2.2.2 pipeline [36], defining as reference genome the longest one thousand scaffolds of the

Spil_haplotype1 genome from the Supernova assembly, which represents about half of the genome (488.5Mb). The LongRanger pipeline was run with default setting beside the vcmode defining gatk v4.0.3.0 as the variant caller and the somatic parameters. The longest phase block was 2.86 Mb and the N50 phase block was 0.476 Mb.

Single nucleotide polymorphisms (SNP's) were furthered filtered to obtain only phased and heterozygous SNP's between the two alleles with a coverage higher than 10-fold using vcftools. A VCF file was obtained containing 2,369,617 filtered SNPs (Additional file 1), in concordance with the estimated mean distance between heterozygous SNPs in the whole genome of 197 bp, by the Supernova input report.

De novo transcriptome assembly

Editing the 596 million paired-end raw reads for contamination (e.g. adapters) was done with the Trim Galore wrapper tool [37], low-quality base trimming with Cutadapt [38] and the output overall quality reports of the edited reads with FastQC [39]. The 553.2 million edited paired-end reads were *de novo* assembled using Trinity v2.5.1 [40] with a minimum contig length of 200 bp, 50x coverage read depth normalization, and RF strand-specific read orientation. The same parameters were used for each of the tissue specific *de novo* assemblies. The genome and transcriptome assemblies were conducted on the National Distributed Computing Infrastructure [41].

The twelve *de novo* transcriptome assemblies (Table 3) were quality assessed with TransRate v1.0.3 [42] for assembly optimization, including 11 tissue-specific assemblies and a mulit-tissue assembly. The multi-tissue assembly with all reads resulted in an assembled transcriptome of 170,478 transcript contigs folloowing the

TransRate step. Functional annotation was performed using the Trinotate pipeline [43] and integrated into a SQLite database. All annotation was based on the best deduced open reading frame (ORF) obtained with the Transdecoder v1.03 [44]. Of the 170,478 transcripts contigs, 27,078 (16%) were inferred to ORF protein sequences. Query of SwissProt (e-value cutoff of 1e-5) via blastx of total contigs resulted in 43,458 (26%) annotated transcripts. The ORFs were queried against SwissProt (e-value cutoff of 1e-5) via blastp and PFAM via HMMER v3.1b2 hmmscan [45] resulting in 19,705 (73% of ORF) and 16.538 (61% of ORF) SwissProt and PFAM annotated contigs respectively. The full annotation report with further functional annotation, such as signal peptides, transmembrane regions, eggnog, Kyoto Encyclopedia of Genes and Genomes (KEGG), and Gene Ontology annotation are listed in tabular format in Additional file 2.

Conclusion

The genomic and transcriptomic resources here reported are important tools for future studies to understand sardine response at the levels of physiology, population and ecology of the causal factors responsible for the recruitment and collapse of the sardine stock in Iberian Atlantic coast. Besides the commercial interest, the sardine has a key trophic level bridging energy from the primary producers to the top predators in the marine ecosystem, and thus disruption of the population equilibrium is likely to reverberate throughout the food chain.

Despite an initial assessment of the sardine genome characteristics indicating a high level of repeats and heterozygosity, which poses a challenge to *de novo* genome assembly, a reasonable draft genome was obtained with the 10X Genomics linked-reads technology. The ability to tag and cluster the reads to individual DNA molecules

has proven to have similar advantages for scaffolding, as long reads technologies such as Nanopore and Pacific Biosciences, but with the advantage of high coverage and low error rates. The advantage for *de novo* genomic assemblies is evident in comparison to simple short read data, especially in the case of wild species with highly heterozygous genomes, resulting in many genomic regions uncaptured and with lower scaffolding yield due to repeated content.

The high heterozygosity identified here hints future problems in monitoring sardine populations using low resolution genetic data. However, the phased SNPs obtained in this study can be used to initiate the development of a SNP genetic panel for population monitoring, with SNPs representative of haplotype blocks, allowing insights into the patterns of linkage disequilibrium and the structure of haplotype blocks across populations.

Availability of the supporting data

Raw data, assembled transcriptomes, and assembled genomes are available at the European Bioinformatics Institute ENA archive with the project accession PRJEB27990. The annotated genome assembly is published in the wiki-style annotation portal ORCAE [34].

Acknowledgements

This research was supported by national funds from FCT - Foundation for Science and Technology through project UID/Multi/04326/2013 and received support of the INCD funded by FCT and FEDER under project 22153-01/SAICT/2016. The EMBRIC configurator service received funding from the European Union’s Horizon 2020

research and innovation programme under grant agreement No 654008. The authors thank Pedro Guerreiro for experimental sampling.

References

1. Parrish RH, Serra R and Grant WS. The monotypic sardines, *Sardina* and *Sardinops* - Their taxonomy, distribution, stock structure, and zoogeography. Can J Fish Aquat Sci. 1989;46 11:2019-36. doi:10.1139/f89-251.
2. Silva A. Morphometric variation among sardine (*Sardina pilchardus*) populations from the northeastern Atlantic and the western Mediterranean. ICES J Mar Sci. 2003;60 6:1352-60. doi:10.1016/S1054-3139(03)00141-3.
3. Lavoue S, Miya M, Saitoh K, Ishiguro NB and Nishida M. Phylogenetic relationships among anchovies, sardines, herrings and their relatives (Clupeiformes), inferred from whole mitogenome sequences. Mol Phylogenet Evol. 2007;43 3:1096-105. doi:10.1016/j.ympev.2006.09.018.
4. Santos AMP, Borges MDF and Groom S. Sardine and horse mackerel recruitment and upwelling off Portugal. ICES J Mar Sci. 2001;58 3:589-96. doi:10.1006/jmsc.2001.1060.
5. Checkley Jr. DM, Asch RG and Rykaczewski RR. Climate, Anchovy, and Sardine. Ann Rev Mar Sci. 2017;9 1:469-93. doi:10.1146/annurev-marine-122414-033819.
6. ICES. *Report of the Working Group on Southern Horse Mackerel, Anchovy and Sardine (WGHANSA), 24–29 June 2017, Bilbao, Spain. CM 2017/ACOM:17, 640 p.* 2017.
7. Atarhouch T, Ruber L, Gonzalez EG, Albert EM, Rami M, Dakkak A, et al. Signature of an early genetic bottleneck in a population of Moroccan sardines

(*Sardina pilchardus*). Mol Phylogenet Evol. 2006;39 2:373-83. doi:10.1016/j.ympev.2005.08.003.

8. Santos MB, Gonzalez-Quiros R, Riveiro I, Cabanas JM, Porteiro C and Pierce GJ. Cycles, trends, and residual variation in the Iberian sardine (*Sardina pilchardus*) recruitment series and their relationship with the environment. ICES J Mar Sci. 2012;69 5:739-50. doi:10.1093/icesjms/fsr186.

9. Leitao F, Alms V and Erzini K. A multi-model approach to evaluate the role of environmental variability and fishing pressure in sardine fisheries. J Mar Syst. 2014;139:128-38. doi:10.1016/j.jmarsys.2014.05.013.

10. Tinti F, Di Nunno C, Guarniero I, Talenti M, Tommasini S, Fabbri E, et al. Mitochondrial DNA sequence variation suggests the lack of genetic heterogeneity in the Adriatic and Ionian stocks of *Sardina pilchardus*. Mar Biotechnol (NY). 2002;4 2:163-72. doi:10.1007/s10126-002-0003-3.

11. Jemaa S, Bacha M, Khalaf G, Dessailly D, Rabhi K and Amara R. What can otolith shape analysis tell us about population structure of the European sardine, *Sardina pilchardus*, from Atlantic and Mediterranean waters? J Sea Res. 2015;96:11-7. doi:10.1016/j.seares.2014.11.002.

12. Boehm JT, Waldman J, Robinson JD and Hickerson MJ. Population genomics reveals seahorses (*Hippocampus erectus*) of the western mid-Atlantic coast to be residents rather than vagrants. PLOS ONE. 2015;10 1:e0116219. doi:10.1371/journal.pone.0116219.

13. Hendricks S, Anderson EC, Antao T, Bernatchez L, Forester BR, Garner B, et al. Recent advances in conservation and population genomics data analysis. Evolutionary Applications. 2018;11 8:1197-211. doi:10.1111/eva.12659.

14. Marcalo A, Guerreiro PM, Bentes L, Rangel M, Monteiro P, Oliveira F, et al. Effects of different slipping methods on the mortality of sardine, *Sardina pilchardus*, after purse-seine capture off the Portuguese Southern coast (Algarve). PLoS One. 2018;13 5:e0195433. doi:10.1371/journal.pone.0195433.

15. UC Davis Bioinformatics Core https://github.com/ucdavis-bioinformatics/proc10xG. Accessed 9/24/2018 2018.

16. Marcais G and Kingsford C. A fast, lock-free approach for efficient parallel counting of occurrences of k-mers. Bioinformatics. 2011;27 6:764-70. doi:10.1093/bioinformatics/btr011.

17. Vurture GW, Sedlazeck FJ, Nattestad M, Underwood CJ, Fang H, Gurtowski J, et al. GenomeScope: fast reference-free genome profiling from short reads. Bioinformatics. 2017;33 14:2202-4. doi:10.1093/bioinformatics/btx153.

18. Weisenfeld NI, Kumar V, Shah P, Church DM and Jaffe DB. Direct determination of diploid genome sequences. Genome Res. 2017;27 5:757-67. doi:10.1101/gr.214874.116.

19. Warren RL. RAILS and Cobbler: Scaffolding and automated finishing of draft genomes using long DNA sequences. J Open Source Soft. 2016;1 7:116. doi:10.21105/joss.00116.

20. Waterhouse RM, Seppey M, Simao FA, Manni M, Ioannidis P, Klioutchnikov G, et al. BUSCO applications from quality assessments to gene prediction and phylogenomics. Mol Biol Evol. 2017;35 3:543-8. doi:10.1093/molbev/msx319.

21. EMBRIC Configurator Service. http://www.embric.eu/node/1371. Accessed 9/24/2018.

22. Smit A and Hubley R: RepeatModeler Open-1.0. http://www.repeatmasker.org (2008). Accessed 9/24/2018.

23. Hubley R, Finn RD, Clements J, Eddy SR, Jones TA, Bao W, et al. The Dfam database of repetitive DNA families. Nucleic Acids Res. 2016;44 D1:D81-9. doi:10.1093/nar/gkv1272.

24. Bao W, Kojima KK and Kohany O. Repbase Update, a database of repetitive elements in eukaryotic genomes. Mob DNA. 2015;6 1:11. doi:10.1186/s13100-015-0041-9.

25. Smit A, Hubley R and Green P. 2013–2015. RepeatMasker Open-4.0. 2013.

26. Holt C and Yandell M. MAKER2: an annotation pipeline and genome-database management tool for second-generation genome projects. BMC Bioinformatics. 2011;12 1:491. doi:10.1186/1471-2105-12-491.

27. Korf I. Gene finding in novel genomes. BMC Bioinformatics. 2004;5 1:59. doi:10.1186/1471-2105-5-59.

28. Keller O, Kollmar M, Stanke M and Waack S. A novel hybrid gene prediction method employing protein multiple sequence alignments. Bioinformatics. 2011;27 6:757-63. doi:10.1093/bioinformatics/btr010.

29. Lomsadze A, Burns PD and Borodovsky M. Integration of mapped RNA-Seq reads into automatic training of eukaryotic gene finding algorithm. Nucleic Acids Res. 2014;42 15:e119. doi:10.1093/nar/gku557.

30. Jones P, Binns D, Chang HY, Fraser M, Li W, McAnulla C, et al. InterProScan 5: genome-scale protein function classification. Bioinformatics. 2014;30 9:1236-40. doi:10.1093/bioinformatics/btu031.

31. Altschul SF, Gish W, Miller W, Myers EW and Lipman DJ. Basic local alignment search tool. J Mol Biol. 1990;215 3:403-10. doi:10.1016/S0022-2836(05)80360-2.

32. Apweiler R, Bairoch A, Wu CH, Barker WC, Boeckmann B, Ferro S, et al. m. Nucleic Acids Res. 2004;32 Database issue:D115-9. doi:10.1093/nar/gkh131.

33. Finn RD, Attwood TK, Babbitt PC, Bateman A, Bork P, Bridge AJ, et al. InterPro in 2017—beyond protein family and domain annotations. Nucleic Acids Res. 2016;45 D1:D190. doi:10.1093/nar/gkw1107.

34. Sardine Genome Annotation Portal. http://bioinformatics.psb.ugent.be/orcae/overview/Spil. Accessed 9/24/2018.

35. Sterck L, Billiau K, Abeel T, Rouze P and Van de Peer Y. ORCAE: online resource for community annotation of eukaryotes. Nat Methods. 2012;9 11:1041. doi:10.1038/nmeth.2242.

36. Zheng GX, Lau BT, Schnall-Levin M, Jarosz M, Bell JM, Hindson CM, et al. Haplotyping germline and cancer genomes with high-throughput linked-read sequencing. Nat Biotechnol. 2016;34 3:303-11. doi:10.1038/nbt.3432.

37. Krueger F: "Trim galore" A wrapper tool around Cutadapt and FastQC to consistently apply quality and adapter trimming to FastQ files. http://www.bioinformatics.babraham.ac.uk/projects/trim_galore/ (2015). Accessed 9/24/2018.

38. Martin M. Cutadapt removes adapter sequences from high-throughput sequencing reads. EMBnet journal. 2011;17 1:10-2. doi:10.14806/ej.17.1.200.

39. Andrews S: FastQC: a quality control tool for high throughput sequence data. http://www.bioinformatics.babraham.ac.uk/projects/fastqc/ (2010). Accessed 9/24/2018.

40. Haas BJ, Papanicolaou A, Yassour M, Grabherr M, Blood PD, Bowden J, et al. De novo transcript sequence reconstruction from RNA-seq using the Trinity platform for reference generation and analysis. Nat Protoc. 2013;8 8:1494-512. doi:10.1038/nprot.2013.084.

41. INCD - National Distributed Computing Infrastructure is a digital infrastructure supporting research, approved within the framework of the strategic research infrastructures of the Science and Technology Foundation (FCT). . http://www.incd.pt/?p=sobre-nos&lang=en. Accessed 9/24/2018.

42. Smith-Unna R, Boursnell C, Patro R, Hibberd JM and Kelly S. TransRate: reference-free quality assessment of de novo transcriptome assemblies. Genome Res. 2016;26 8:1134-44. doi:10.1101/gr.196469.115.

43. Bryant DM, Johnson K, DiTommaso T, Tickle T, Couger MB, Payzin-Dogru D, et al. A Tissue-Mapped Axolotl De Novo Transcriptome Enables Identification of Limb Regeneration Factors. Cell Rep. 2017;18 3:762-76. doi:10.1016/j.celrep.2016.12.063.

44. TransDecoder identifies candidate coding regions within transcript sequences. http://github.com/TransDecoder. Accessed 9/24/2018.

45. Finn RD, Clements J and Eddy SR. HMMER web server: interactive sequence similarity searching. Nucleic Acids Res. 2011;39 Web Server issue:W29-37. doi:10.1093/nar/gkr367.

Tables

Table 1. List of descriptive metrics estimated by Supernova on the input sequence data for the *de novo* genome assembly.

Number of paired reads used	607.36 M
Mean read length after trimming	138.50 bp
Median insert size	345 bp
Weighted mean DNA molecule size	46.41 Kb
N50 reads per barcode	612
Raw coverage	78.35 X
Effective read coverage	52.91 X
Mean distance between heterozygous SNPs	197 bp

Table 2. Descriptive metrics of genome assemblies, the two haploids genomes Spil_haploid1 (ERZ724592) and Spil_haploid2 (ERZ724593) assembled/scaffolded solely by Supernova and the consensus genome Spil (GCA_900492735.1) assembled/scaffolded by Supernova plus Rails/Cobbler.

Scaffolds	Spil_haploid1	Spil_haploid2	Spil
Largest	6 835 195 bp	6 849 541 bp	6 843 175 bp
Number			
>=100Kb	874	872	890
>= 10Kb	8 301	8 298	8 760
>= 1Kb (total)	117 698	117 698	117 259
L50 / N50			
>=100Kb	135 / 905 971 bp	134 / 925 166 bp	137 / 899 108 bp
>= 10Kb	242 / 572 700 bp	242 / 568 166 bp	254 / 552 199 bp
>= 1Kb	859 / 102 905 bp	860 / 102 672 bp	903 / 96 617 bp
Assembly size			
>=100Kb	469 371 101 bp	468 838 424 bp	473 549 829 bp
>= 10Kb	622 164 859 bp	621 688 061 bp	636 490 596 bp
>= 1Kb	935 547 786 bp	935 081 460 bp	949 618 126 bp

Table 3 – Summary statistics of generated transcriptome data for the eleven tissues.

Tissue	Paired raw reads	Contigs	CDS deduced	SwissProt annotated	Accession number
Gill/Branchial Arch	29 783 994	62 526	29.3%	38.6%	ERS2629269
Liver	33 479 471	53 104	29.7%	40.1%	ERS2629273
Spleen	25 634 530	66 419	31.6%	40.4%	ERS2629276
Ovary	22 241 327	42 521	38.1%	42.5%	ERS2629270
Midgut	28 016 117	75 782	31.0%	39.5%	ERS2629274
White Muscle	24 409 160	49 266	35.4%	44.8%	ERS2629277
Red Muscle	30 653 774	55 873	30.3%	42.1%	ERS2629275
Kidney	27 861 879	59 495	30.8%	37.3%	ERS2629272
Head Kidney	25 280 960	65 888	32.2%	38.4%	ERS2629271
Brain/Pituitary	24 467 352	75 620	24.5%	37.1%	ERS2629267
Caudal Fin (Skin/Cartilage/Bone)	26 342 097	64 832	23.9%	38.0%	ERS2629268
All Tissues	298 170 661	170 478	15.9%	25.5%	ERS2629362

Figure legends

Figure 1. European sardine (photo credit ©Citron / CC BY-SA 3.0)

Figure 2. 23-mer depth distribution to estimate genome size (907Mb), repeat content (40.7%) and heterozygosity level (1.43%). Two kmer coverage peaks are observed at 28X and 50X.

Additional files

Additional file 1. Heterozygous SNPs identified in the phased haploid blocks listed in a VCF file format.

Additional file 2. Annotation of all tissues transcriptome assembly in a tabular format.

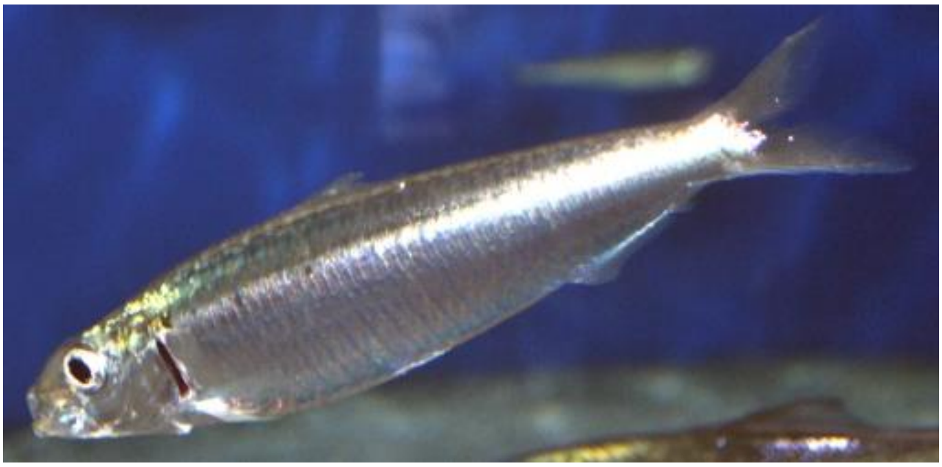

Figure 1.

Click here to download Figure Louro_et_al_Figure2.docx

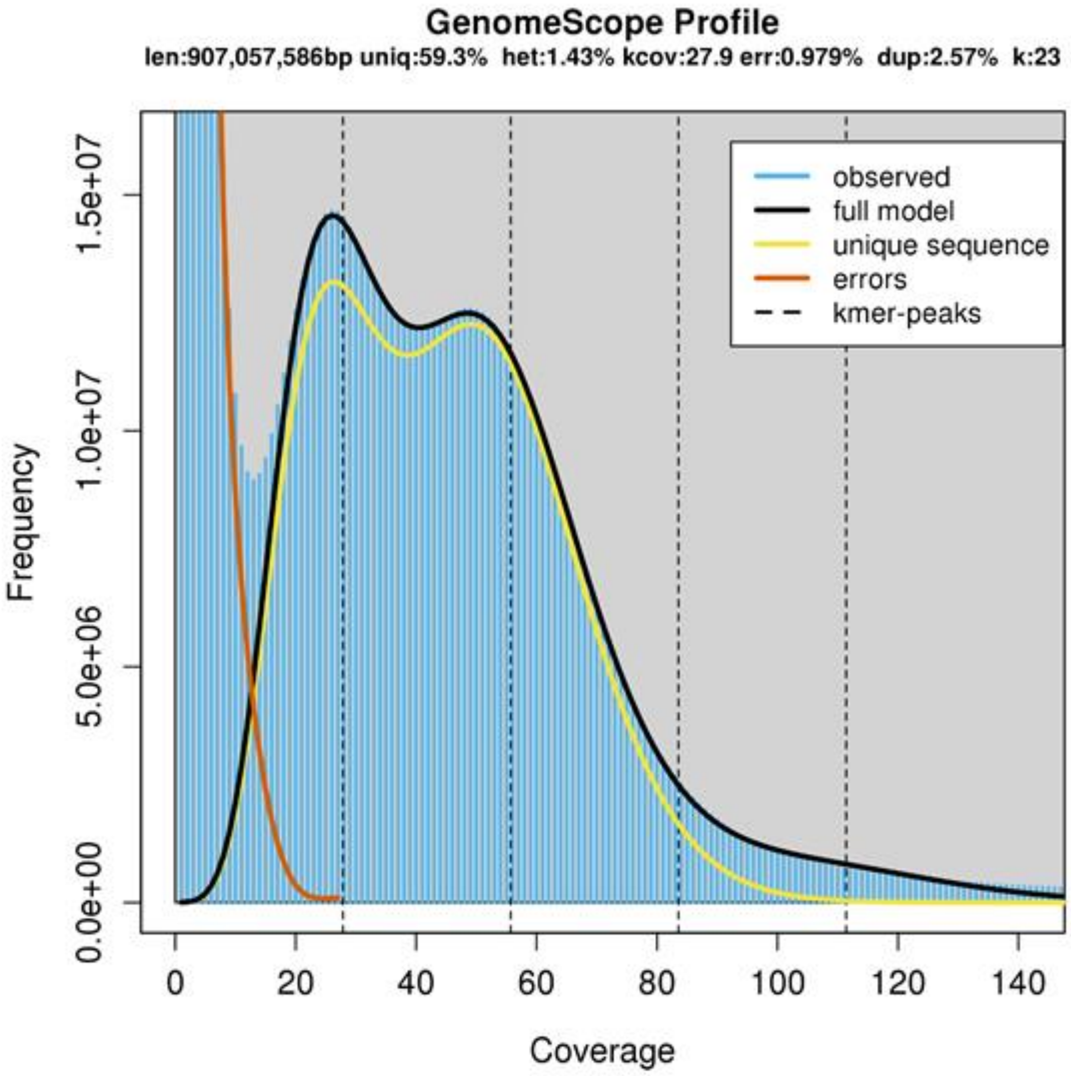

Figure 2.

additional file 1

Click here to access/download

Supplementary Material

Spil_SNP_phased_COV10_nbc.vcf

additional file 2

Click here to access/download
Supplementary Material
additional_file_2.txt

Cover Letter

September 24, 2018

Dear Editor,

We would like to submit the manuscript entitled "**A haplotype-resolved draft genome of the European sardine (*Sardina Pilchardus*)**" by Louro *et al.* for publication in GigaScience as a Data Note article. In the manuscript we report the first assembled and annotated draft genome of the European sardine. We report three de-novo assemblies, a consensus draft genome (size 950Mbp; N50 length 97 Kb) and two haploid-resolved draft genomes (size 935 Mbp; N50 length 103 Kb), made possible by the use 10X Genomics linked-reads technology. Phased sequencing also allowed the variant calling between phased alleles resulting in more than 2.3 million SNPs with heterozygous loci identified. The transcriptomes of eleven tissues were also de-novo assembled and used to aid the functional annotation of the genome resulting in 29,408 genes predicted.

This resource will be important to foster development of omics approaches to resource conservation and fisheries management a species with cultural and economic value. Consequently, the sequences have already been made available to the public. We therefore expect that you will consider the manuscript suitable for publication in GigaScience.

All authors have approved the manuscript for submission and state that the content of the manuscript has not been published, or submitted for publication elsewhere. The authors also declare that no potential competing interests or any issues relating to journal policies exists.

Yours sincerely,

Adelino Canário
