# Supplementary material for: A haplotype-resolved draft genome of the European sardine (Sardina pilchardus)
Source: Gigascience. 2019 May 21;8(5):giz059. doi: 10.1093/gigascience/giz059 (PMC6528745; doi:10.1093/gigascience/giz059)
Supplement: Supplemental Files [file giz059_supplemental_files.zip › additional_file_3.docx]

Table S1. Sequence alignment statistics of the 97 proteins concatenated for the phylogenetics analyses. The concatenated sequence alignment of the 97 proteins contained 14,515 sites without gaps of which 7391 were constant, 7123 variable, and 3879 parsimony informative.

|  | **Alignment sites** | | | | |
| --- | --- | --- | --- | --- | --- |
| **Gene Name** | **total** | **constant** | **variable** | **ambiguous** | **pars_infor** |
| cmtr1 | 139 | 64 | 75 | 0 | 36 |
| psmd6 | 384 | 295 | 89 | 0 | 40 |
| nme4 | 120 | 18 | 102 | 0 | 52 |
| ddx18 | 379 | 281 | 98 | 0 | 62 |
| atp6v0d1 | 213 | 185 | 28 | 0 | 5 |
| trappc3 | 99 | 73 | 26 | 0 | 18 |
| tmem64 | 20 | 2 | 18 | 0 | 10 |
| dera | 276 | 155 | 121 | 9 | 85 |
| wdr45 | 199 | 130 | 69 | 0 | 27 |
| tsn | 165 | 68 | 97 | 1 | 31 |
| glo1 | 154 | 100 | 54 | 0 | 28 |
| jmjd7 | 85 | 37 | 48 | 0 | 32 |
| naa20 | 128 | 92 | 36 | 0 | 8 |
| mrpl46 | 53 | 13 | 40 | 0 | 27 |
| tfb2m | 176 | 17 | 159 | 0 | 104 |
| cox11 | 183 | 118 | 65 | 0 | 38 |
| med10 | 79 | 46 | 33 | 0 | 13 |
| cactin | 221 | 150 | 71 | 0 | 35 |
| rab20 | 118 | 50 | 68 | 81 | 35 |
| hectd3 | 425 | 74 | 351 | 17 | 119 |
| nanp | 175 | 47 | 128 | 0 | 95 |
| gba2 | 349 | 143 | 206 | 0 | 113 |
| mccc2 | 119 | 92 | 27 | 0 | 13 |
| hmces | 45 | 15 | 30 | 0 | 15 |
| suclg1 | 271 | 219 | 52 | 0 | 38 |
| coq9 | 190 | 46 | 144 | 0 | 68 |
| ube2g2 | 86 | 74 | 12 | 0 | 5 |
| yipf4 | 58 | 47 | 11 | 0 | 5 |
| zgc | 81 | 29 | 52 | 0 | 25 |
| glod4 | 213 | 53 | 160 | 0 | 70 |
| si | 89 | 36 | 53 | 0 | 31 |
| fam120b | 64 | 18 | 46 | 0 | 32 |
| tbc1d7 | 173 | 64 | 109 | 0 | 72 |
| washc3 | 56 | 28 | 28 | 0 | 16 |
| phb | 255 | 178 | 77 | 0 | 33 |
| slc35b2 | 210 | 144 | 66 | 0 | 40 |
| ddost | 414 | 220 | 194 | 0 | 76 |
| sharpin | 68 | 22 | 46 | 1 | 27 |
| ndufa6 | 122 | 46 | 76 | 0 | 42 |
| gemin2 | 16 | 1 | 15 | 0 | 6 |
| gemin8 | 100 | 38 | 62 | 18 | 42 |
| sspn | 40 | 7 | 33 | 0 | 24 |
| atp6v1d | 243 | 175 | 68 | 0 | 39 |
| rxylt1 | 80 | 27 | 53 | 0 | 37 |
| mrpl51 | 64 | 24 | 40 | 0 | 31 |
| bloc1s4 | 81 | 29 | 52 | 0 | 24 |
| apex1 | 277 | 152 | 125 | 0 | 72 |
| cgrrf1 | 83 | 12 | 71 | 0 | 57 |
| sra1 | 72 | 18 | 54 | 0 | 32 |
| enoph1 | 149 | 47 | 102 | 0 | 52 |
| slc38a9 | 238 | 106 | 132 | 0 | 71 |
| dcaf12 | 135 | 87 | 48 | 0 | 20 |
| hspa13 | 17 | 11 | 6 | 2 | 3 |
| mrps10 | 113 | 43 | 70 | 0 | 40 |
| psmb3 | 147 | 105 | 42 | 0 | 8 |
| mlst8 | 271 | 213 | 58 | 0 | 17 |
| timm29 | 179 | 39 | 140 | 0 | 106 |
| pak1ip1 | 138 | 32 | 106 | 0 | 71 |
| exoc8 | 570 | 335 | 235 | 0 | 134 |
| setd4 | 39 | 17 | 22 | 0 | 11 |
| pex2 | 51 | 21 | 30 | 51 | 22 |
| FAM109B | 128 | 54 | 74 | 0 | 33 |
| txndc5 | 304 | 107 | 197 | 0 | 109 |
| phax | 98 | 32 | 66 | 0 | 45 |
| TIMM21 | 24 | 6 | 18 | 0 | 4 |
| ndufv2 | 119 | 43 | 76 | 1 | 35 |
| gtf2h3 | 173 | 108 | 65 | 0 | 34 |
| ttc36 | 170 | 72 | 98 | 0 | 67 |
| rnaseh2a | 48 | 21 | 27 | 0 | 18 |
| cmpk2 | 135 | 38 | 97 | 0 | 56 |
| tsku | 138 | 49 | 89 | 0 | 56 |
| abhd13 | 279 | 130 | 149 | 0 | 67 |
| mrpl32 | 30 | 13 | 17 | 0 | 13 |
| acot13 | 111 | 47 | 64 | 0 | 32 |
| gclm | 191 | 78 | 113 | 0 | 68 |
| gatad1 | 49 | 30 | 19 | 0 | 11 |
| clpp | 173 | 130 | 43 | 0 | 22 |
| ndufaf1 | 137 | 45 | 92 | 0 | 61 |
| mtfr1 | 32 | 10 | 22 | 0 | 11 |
| cdkn2aipnl | 50 | 22 | 28 | 0 | 20 |
| crnkl1 | 642 | 445 | 197 | 0 | 126 |
| ap2s1 | 134 | 131 | 3 | 0 | 2 |
| serpine2 | 138 | 19 | 119 | 0 | 76 |
| med29 | 127 | 80 | 47 | 0 | 21 |
| sap30l | 96 | 84 | 12 | 0 | 4 |
| scnm1 | 44 | 16 | 28 | 0 | 17 |
| mrps25 | 131 | 53 | 78 | 0 | 46 |
| ube2s | 107 | 30 | 77 | 0 | 13 |
| zcchc17 | 48 | 16 | 32 | 0 | 19 |
| srp19 | 118 | 50 | 68 | 0 | 35 |
| llph | 90 | 40 | 50 | 0 | 37 |
| slc35b4 | 157 | 77 | 80 | 0 | 34 |
| hypk | 66 | 47 | 19 | 0 | 8 |
| mcee | 91 | 56 | 35 | 0 | 20 |
| glrx5 | 88 | 56 | 32 | 0 | 20 |
| ecsit | 189 | 78 | 111 | 0 | 67 |
| gosr2 | 102 | 50 | 52 | 0 | 32 |
| Concatenated | 14514 | 7391 | 7123 | - | 3879 |
