# Supplementary material for: A haplotype-resolved draft genome of the European sardine (Sardina pilchardus)
Source: Gigascience. 2019 May 21;8(5):giz059. doi: 10.1093/gigascience/giz059 (PMC6528745; doi:10.1093/gigascience/giz059)
Supplement: Response_to_Reviewer_Comments_Original_Submission.pdf [file giz059_response_to_reviewer_comments_original_submission.pdf]

Author's Response To Reviewer Comments

Close

##
Editor:
#1: To put this work in context and offer sufficient validation please provide comparisons in quality and a phylogenomic tree with related sequenced fish species. This can include the other recently sequenced sardine genomes. The referees (and if published, readers) can then judge the quality, utility and context better.
Reply 1: A section with the phylogenetic analysis and tree was added. A quality statistic metrics comparison with the other published sardine genome assembly (Machado et al, 2008) was also added (table 2) and discussed. An orthologous proteome analysis was also added.

#2: Please include other identifiers and accessions (fishbase and NCBI taxon IDs, ORCIDs, RRIDs, sample and data accessions) for reproducibility purposes, and make sure there is sufficient methodological detail.
Reply 2: All missing identifiers/accessions were added. The few software that don't have RRIDs are referenced and version indicated. Assembly data accession references are now clearer.

Reviewer reports:
Reviewer #1: In this manuscript, the authors report a draft genome for the critically important European Sardine For the most part, the approach is well thought-out and justified, but there are some key concerns involving findings and methodology.

#3: For example, the annotation pipeline is unclear. In line 169, it is described as a custom pipeline, but there is no detail regarding how specifically MAKER is used to annotate the genome.
Reply 3: "Custom" pipeline wasn't the best choice of words as it gave the idea of an in-house pipeline, which is not the case. Maker is an established annotation software that pipes several other programs (http://www.yandell-lab.org/software/maker.html). We now added a detailed description of the maker workflow and respective software used to annotate the genome (line 198):
"The Maker v2.31.10 (MAKER, RRID:SCR_005309) [29] pipeline was used iteratively (five times) to annotate the SP_G consensus genome. The annotations generated in each iteration were kept in the succeeding annotation steps and in the final General Feature Format (GFF) file. During the first Maker run the already described de novo transcriptome was mapped to the genome using blastn v2.7.1 (BLASTN, RRID:SCR_001598) [30] (est2genome parameter in Maker). Moreover, the repetitive elements found with RepeatMasker were used in the Maker pipeline. This initial gene models created by Maker were then used to train Hidden Markov Model (HMM) based gene predictors. The preliminary GFF file generated by this first iteration run was used as input to train SNAP v2006-07-28 [31]. Using the scripts provided directly by Maker (maker2zff) and SNAP (fathom, forge and hmm-assembler.pl) an HMM file was created and used as input for the next Maker iteration (snaphmm option in maker configuration file). For the next iteration, the gene-finding software Augustus v3.3 (Augustus, RRID:SCR_008417) [32] was self-trained running BUSCO with the specific parameter (--long), that turn on the Augustus optimization mode for self-training. The resulted predicted species model from Augustus was included in the pipeline in the third Maker run. For the fourth iteration, GeneMark-ES v4.32 (GeneMark, RRID:SCR_011930) [33], a self-training gene prediction software, was executed and the resulting HMM file was integrated into the Maker pipeline. As further evidence for the annotation, in the last run of maker, the genome was queried using blastx v2.7.1 (BLASTX, RRID:SCR_001653) (protein2genome parameter in Maker),against the deduced proteomes of herring (GCF_000966335.1), (Clupea harengus, NCBI:txid7950, Fishbase ID:24) zebrafish (Danio rerio, NCBI:txid7955, Fishbase ID:4653) (GCF_000002035.6), blind cave fish (Astyanax mexicanus, NCBI:txid7994, Fishbase ID:2740) (GCF_000372685.2), European sardine [34] and all proteins from teleost fishes in the UniProtKB/Swiss-Prot database (UniProtKB, RRID:SCR_004426) [35]. After the five Maker runs the selected 40 777 genes models are the ab initio predictions supported by the transcriptome and proteome evidence."

#4: There is no mention of how evidence such as transcriptome evidence from the sardine or other species was used to annotate via MAKER.

Reply 4: The use of sardine transcriptome is now better explained in the maker workflow description (see above reply 3).
Other species were used in the annotation for protein evidence, while for transcriptome evidence we used solely the transcriptome from the sardine. The transcriptome generated was quite extensive and representative of the adult stage of the sardine.

#5: In addition, it is stated in line 183 that 17,199 (65%) proteins received functional annotation. This seem like low efficiency as over a third of potential genes remain unannotated even though there is a wealth of protein sequence data available from fish genomes. This could be due to gene prediction calling a large number of false positives, but it is hard to interperet based on the brief explanation of a custom pipeline.
Reply 5: We have revised the annotation procedure using the Maker annotation pipeline. We did an extra Maker iterative run (Protein2genome) leading to an improved genome annotation with more gene models and with better AED score (median 0.16, data not shown). With this improvement we are now able to functionally annotate 95.3% of the predicted gene coding proteins. Many of the false positive predicted genes were eliminated because now we have the ab initio predictions supported by both protein and transcriptome evidences.

#6: There are other unjustified cuttoffs such as the fact that only half of the genome was used for some analyses (line 190).
Reply 6: The reason for the cuttoff is that the LongRanger software has a maximum input of 1000 scaffolds as reference genome. We have rewritten the sentence (line 261):
"FASTQ files were processed using the 10x Genomics LongRanger v2.2.2 pipeline [41] with a maximum input limit of one thousand scaffolds, defined as reference genome, and representing about half of the genome size (488.5 Mb)."

#7: The authors mention that the genome contains high heterozygosity, but offer no point of reference or comparison to other species so that it is demonstrated to be high.
Reply 7: A sentence addressing this point was added to the manuscript line 274: "This high SNP heterozygosity (1/206), observed solely in the comparison of the phased alleles, is higher than the average nucleotide diversity of the previously reported marine fish of wild populations: 1/390 in yellow drum [44], 1/309 in herring [45], 1/435 in coelacanth [46], 1/500 in cod [47] and 1/700 in stickleback [48]."

#8: Lastly, there is little discussion about the transcriptomes and how they were used for the genome analyses.
Reply 8: The transcriptome was used for the genome annotation and is now better described and discussed (reply 3).
In addition, the results of the UTR gene prediction based on the transcriptomes is now presented in the following added sentence (line 225): "Based on the transcriptomic evidence, 12 761 gene models were annotated with untranslated regions (UTR) features, more specifically 9 486 gene models with either 5' or 3' UTR and 3 275 gene models with both UTR features."

9: This is also an awesome resource that is established by this study, but it needs more attention in the manuscript. A commented manuscript is attached.
Reply 9: The comments in the manuscript have all been dealt with and corrections made:

PDF-# 9.1: this sentence is a bit unclear. Regarding previous sentence: "A VCF file was obtained containing 2,369,617 filtered SNPs (Additional file 1), in concordance with the estimated mean distance between heterozygous SNPs in the whole genome of 197 bp, by the Supernova input report."
Reply 9.1: This sentence was rewritten for clarity (line 270): "A VCF file was obtained containing 2 369 617 filtered SNPs (Additional file 1) resulting in a mean distance between heterozygous phased SNPs of 206 bp. Similar results were obtained in the Supernova input report estimation (Table 1) of mean distance between heterozygous SNPs in the whole genome of 197 bp."

PDF-# 9.2: This custom pipeline needs elaboration and description. It is a black box. For example, how was protein homology assessed? What did you use as evidence for annotation? sardine transcriptomes? proteins from other fishes?
Reply 9.2: see replies 3 and 4.

PDF-# 9.3: This seems like a low number, and should be addressed in the discussion. What about the other 35%? It seems like the ab initio gene prediction might be calling a lot of false positives. However,

it is difficult to see how evidence was used in the pipeline to identify protein coding genes.
Reply 9.3: see replies 3 and 4.

PDF-# 9.4: how were they asses with TransRate?
Reply 9.4: The following was added (line 293): "…with read evidence for assembly optimization, by specifying the contigs fasta file and respective left and right edited reads to be mapped."

PDF-# 9.5: This should be described in the above paragraph. Regarding the information: "… including 11 tissue-specific assemblies and a mulit-tissue assembly."
Reply 9.5: The sentence in the previous paragraph was edited to: "The same parameters were used for each of the 11 tissue-specific de novo assemblies."

PDF-# 9.6: why such a low number? Regarding "Of the 170,478 transcripts contigs, 27,078 (16%) were inferred to ORF protein sequences."
Reply 9.6: The values could be explained based on several reasons: 1) the de novo contigs may represent all types of expression products, such as non-coding RNA, by products of mRNA processing (eg, intron cleavage) or even artefacts of the de novo assembly. From our experience in transcriptome assembly, the bigger the input of RNAseq reads (553 M edited reads) the higher the number of assembled contigs (170 478) that contain non-coding products. The ORF number (27 078) is closer to the expected amount of coding expression products.

PDF-# 9.7: again why such a reduction in confirmed transcripts? Regarding "Query of SwissProt (e-value cutoff of 1e-5) via blastx of total contigs resulted in 43,458 (26%) annotated transcripts."
Reply 9.7: see reply 9.6.

PDF-# 9.8: how do we know the heterozygosity is high? What are we comparing it to?
Reply 9.8: A sentence was added to address this point (line 275): "This high SNP heterozygosity (1/206), observed solely in the comparison of the phased alleles, is higher than the average nucleotide diversity of the previously reported marine fish of wild populations: 1/390 in yellow drum [44], 1/309 in herring [45], 1/435 in coelacanth [46], 1/500 in cod [47] and 1/700 in stickleback [48]."

10: Figure 1 is also blurry and it is difficult to see the head region of the fish.
Reply 10: We have replaced figure 1.

11: Overall, more detail and justification is needed for methods and results, and the study would benefit by a comparison or the use of available data from other fish genomes. If these changes are implemented, the study would provide an excellent resource for a valuable fishery.
Reply 11: All suggested changes have been made. We thank the reviewer for valuable remarks which we greatly improved the manuscript.

##
Reviewer #2: The authors of this manuscript report the sequencing of the Europe sardine genome and transcriptome data of selected tissues. Although the obtained resources are novel and valuable, the manuscript does not provide sufficient data to validate their reliability and utility.
#12: The 'Conclusion' part of the Abstract does not provide any conclusion from this study.
Reply 12: The abstract has been modified to address this remark: "A draft genome was obtained with the 10X Genomics linked-reads technology, despite a high level of sequence repeats and heterozygosity that are expected genome characteristics of a wild sardine. The reference sardine genome and respective variant data are a cornerstone resource of ongoing population genomics studies to be integrated into future sardine stock assessment modelling to better manage this valuable resource."

13: The epithet of the species name in the title ('Pilchardus') should not be capitalized.
Reply 13: This typo has been corrected.

14: In Abstract: 'Two haploid and a consensus draft genomes were assembled, with a total size of 935 Mbp (N50 103 Kb) and 950Mbp (N50 97 Kb), respectively.' - it is confusing to distinguish which length stats is applied to which genome assembly, in this sentence.
Reply 14: This sentence has now been rephrased: "Three draft genomes were assembled: two haploid genomes with a total size of 935 Mbp (N50 103Kb) each, and a consensus genome with a total size of 950 Mbp (N50 97Kb)."

15: In the public database NCBI Assembly, I have found two genome assemblies for this species,

whose IDs are SP_G and UP_Spi. It is not clear to me which of these corresponds to the Illumina-based or the Chromium-based assembly in the manuscript. The authors need to sort out this problem and present their correspondences in a more clear-cut way.
Reply 15: We submitted our assemblies to the ENA archive project PRJEB27990, with the accession number of the three assemblies GCA_900499035.1 (consensus assembly), UOTT01000000 (haplotype1), and UOTU01000000 (haplotype2). The consensus assembly (SP_G) the reviewer accessed in the NCBI public database was synchronized automatically with ENA. The UP_Spi is a genome draft assembly submitted soon after from another study by other authors (Machado et al, 2018). All ID accessions are now clearly described in the manuscript. At the time of our manuscript submission neither the other genome (UP_Spi) nor the corresponding publication was available. Now we cite and compare the assemblies from the two studies (Table 2).

16: The composition of the two genome assemblies in NCBI Assembly differs particularly in the length of the shortest sequence (200bp vs 1000bp) which can largely affect other length-based metrics, including the N50 scaffold length. I wonder what the authors' policy behind this variable length cut-off was, and also how they describe it in the manuscript. If the authors did not have any coherent policy, they should reconsider this point and revise the manuscript and the genome assemblies in the NCBI database.
Reply 16: Only the consensus assembly of our study is present in NCBI, no filtering was performed on the contigs/scaffolds to inflate the N50. The Supernova default was 1000bp contig minimum size.

17: Also, in the genome assemblies available at NCBI Assembly, I observed a weird distribution of the lengths of 'N' tracts (stretches of undetermined bases) - they are all round numbers for SP_G, while 'N' tracts with the length of 20 is the majority. I wonder whether the authors noticed these, and think that it is worth reasoning possible causes.
Reply 17: We did noticed such behaviour from the Supernova assembler reflected in the pseudohaplotypes output assemblies. The simple explanation is Supernova is able to estimate the gap size based on barcodes spanning the gaps, i.e gaps have linkage evidence through the barcodes linking reads to DNA molecules, and not solely gaps based on reads pairs. Further detailed explanation can be found in Supernova publication (https://www.biorxiv.org/content/early/2016/08/19/070425) in particular at "Supplemental Note 5. Supernova gap size estimation."
We now include the “N per 100Kb” in table 2 and discuss the issue starting at line 164.

18: For completeness assessment of the genome assemblies they obtained, the authors used the eukaryote ortholog set as well as the Actinopterygii ortholog set. I wonder why the former was used, instead of the vertebrate or metazoan ortholog set. Also, in describing the numbers of orthologs retrieved by BUSCO, the authors should clearly state which category, namely, complete, fragmented, or missing.
Reply 18: We had used the eukaryote ortholog set as a substitution of the core genes CEGMA representation. Following your recommendation, we now present the BUSCO results using the Metazoan ortholog set to represent the coverage of core genes. Following the BUSCO user guide, we also present the results of “actinopterygii” ortholog set as the most related lineage to the sardine.
The results now include all the information requested such as complete, single copy, duplicated, fragmented and missing genes, in the following paragraph:
“The genome completeness assessment was estimated with Benchmarking Universal Single-copy Orthologs (BUSCO) v3.0.1 (BUSCO, RRID:SCR_015008) [23]. The genome was queried (options -m geno –sp zebrafish) against the “metazoa.odb9” lineage set containing 978 orthologs from sixty-five eukaryotic organisms to assess the coverage of core eukaryotic genes, and against the “actinopterygii.odb9” lineage set containing 4584 orthologs from 20 different ray-finned fish species as the most taxon-specific lineage available for the sardine. Using the metazoan odb9 database, 95.4% of the genome had significant matches: 84.5% were complete genes (76.7% single-copy genes and 9.8% duplicates) and 8.9% were fragmented genes. By contrast, using the actinopterygii odb9 database, 84.2% (76.0% complete genes and 8.2% fragmented) had a match, with 69.3% of genes occurring as single copy and 6.7% as duplicates.”

19: Because Figure 2 seems to completely rely on the tool GenomeScope, the authors should cite its source at least in its legend.
Reply 19: This reference is now also added in the figure 2 legend.

We thank the reviewer for valuable remarks which we greatly improved the manuscript.

Close
