# Supplementary material for: A haplotype-resolved draft genome of the European sardine (Sardina pilchardus)
Source: Gigascience. 2019 May 21;8(5):giz059. doi: 10.1093/gigascience/giz059 (PMC6528745; doi:10.1093/gigascience/giz059)
Supplement: Response_to_Reviewer_Comments_Revision_1.pdf [file giz059_response_to_reviewer_comments_revision_1.pdf]

Author's Response To Reviewer Comments

Close

Reply to reviewer reports:

Reviewer #1: The manuscript is much improved and the annotation appears much better. All concerns appear to be addressed. One remaining question I have is how the 97 orthologs were identified to build the phylogeny. Did these come from the Orthofinder analysis? These methods to be elaborated on to show they are true orthologs. Otherwise the manuscript seems ready for publication.

Reply Reviewer #1: The one-to-one ortholog clusters that we used to conduct the phylogenetic analyses were those assembled by Machado et al [20] to which we added the orthologous genes from our genome assembly as identified via HMMER.
To make it clearer we modified the text (lines 310-315) to:
"We conducted a phylogenetic analysis of ray-finned fish (Actinopterygii) taxa based on 17 fish species. The sardine protein data set used in the phylogenetic analysis was obtained by querying the deduced proteins from our sardine genome against the one-to-one orthologous cluster dataset (106 proteins from 17 species) obtained from [20].
For the query, gene models were constructed for each protein with hmmbuild (HMMER v3.1b2) [53] using default options and the orthologous genes from the deduced sardine proteome were searched using hmmsearch (HMMER) with an e-value cuttoff of 10e-3."

We don't describe the clustering methods used by Machado et al [20] to assemble the one-to-one ortholog clusters as we did not repeat those analyses ourselves.

##

Reviewer #2: I see substantial improvements in the manuscript. One remaining issue is the quality of figures. The authors need to present the names of the species with consistency between Figuren 3 and 4. Also, in Figure 4, the name of the main study species in this manuscript 'Sardine' should be consistently included in its Latin species name, Sardina pilchardus.

Reply Reviewer #2: We modified and improved the quality of Figures 3 and 4. In both figures the species names are explicit in italic.

Close
