# Supplementary material for: A haplotype-resolved draft genome of the European sardine (Sardina pilchardus)
Source: Gigascience. 2019 May 21;8(5):giz059. doi: 10.1093/gigascience/giz059 (PMC6528745; doi:10.1093/gigascience/giz059)
Supplement: Reviewer_1_Report_Original_Submission -- Andrew Thompson — 10/29/2018 Reviewed [file giz059_reviewer_1_report_original_submission.pdf]

Reviewer Report

Title: A haplotype-resolved draft genome of the European sardine (Sardina pilchardus)

Version: Original Submission **Date:** 10/29/2018

Reviewer name: Andrew Thompson

Reviewer Comments to Author:

In this manuscript, the authors report a draft genome for the critically important European Sardine For the most part, the approach is well thought-out and justified, but there are some key concerns involving findings and methodology. For example, the annotation pipeline is unclear. In line 169, it is described as a custom pipeline, but there is no detail regarding how specifically MAKER is used to annotate the genome. There is no mention of how evidence such as transcriptome evidence from the sardine or other species was used to annotate via MAKER. In addition, it is stated in line 183 that 17,199 (65%) proteins received functional annotation. This seem like low efficiency as over a third of potential genes remain unannotated even though there is a wealth of protein sequence data available from fish genomes. This could be due to gene prediction calling a large number of false positives, but it is hard to interperet based on the brief explanation of a custom pipeline. There are other unjustified cuttoffs such as the fact that only half of the genome was used for some analyses (line 190). The authors mention that the genome contains high heterozygosity, but offer no point of reference or comparison to other species so that it is demonstrated to be high. Lastly, there is little discussion about the transcriptomes and how they were used for the genome analyses. This is also an awesome resource that is established by this study, but it needs more attention in the manuscript. A commented manuscript is attached. Figure 1 is also blurry and it is diffucult to see the head region of the fish.
Overall, more detail and justification is needed for methods and results, and the study would benefit by a comparison or the use of available data from other fish genomes. If these changes are implemented, the study would provide an excelent resource for a valuable fishery.

Level of Interest

Please indicate how interesting you found the manuscript: Choose an item.

Quality of Written English

Please indicate the quality of language in the manuscript: Choose an item.

Declaration of Competing Interests

Please complete a declaration of competing interests, considering the following questions:

- Have you in the past five years received reimbursements, fees, funding, or salary from an organisation that may in any way gain or lose financially from the publication of this manuscript, either now or in the future?
- Do you hold any stocks or shares in an organisation that may in any way gain or lose financially from the publication of this manuscript, either now or in the future?
- Do you hold or are you currently applying for any patents relating to the content of the manuscript?
- Have you received reimbursements, fees, funding, or salary from an organization that holds or has applied for patents relating to the content of the manuscript?
- Do you have any other financial competing interests?
- Do you have any non-financial competing interests in relation to this paper?

If you can answer no to all of the above, write 'I declare that I have no competing interests' below. If your reply is yes to any, please give details below.

I declare that I have no competing interests

I agree to the open peer review policy of the journal. I understand that my name will be included on my report to the authors and, if the manuscript is accepted for publication, my named report including any attachments I upload will be posted on the website along with the authors' responses. I agree for my report to be made available under an Open Access Creative Commons CC-BY license (http://creativecommons.org/licenses/by/4.0/). I understand that any comments which I do not wish to be included in my named report can be included as confidential comments to the editors, which will not be published.

I agree to the open peer review policy of the journal

To further support our reviewers, we have joined with Publons, where you can gain additional credit to further highlight your hard work (see: https://publons.com/journal/530/gigascience). On publication of this paper, your review will be automatically added to Publons, you can then choose whether or not to claim your Publons credit. I understand this statement.

Yes Choose an item.
