# Supplementary material for: A haplotype-resolved draft genome of the European sardine (Sardina pilchardus)
Source: Gigascience. 2019 May 21;8(5):giz059. doi: 10.1093/gigascience/giz059 (PMC6528745; doi:10.1093/gigascience/giz059)
Supplement: Reviewer_2_Report_Original_Submission -- Shigehiro Kuraku — 11/4/2018 Reviewed [file giz059_reviewer_2_report_original_submission.pdf]

Reviewer Report

Title: A haplotype-resolved draft genome of the European sardine (Sardina pilchardus)

Version: Original Submission **Date:** 11/4/2018

Reviewer name: Shigehiro Kuraku

Reviewer Comments to Author:

The authors of this manuscript report the sequencing of the Europe sardine genome and transcriptome data of selected tissues. Although the obtained resources are novel and valuable, the manuscript does not provide sufficient data to validate their reliability and utility.
The 'Conclusion' part of the Abstract does not provide any conclusion from this study.
The epithet of the species name in the title ('Pilchardus') should not be capitalized.
In Abstract: 'Two haploid and a consensus draft genomes were assembled, with a total size of 935 Mbp (N50 103 Kb) and 950Mbp (N50 97 Kb), respectively.' - it is confusing to distinguish which length stats is applied to which genome assembly, in this sentence.
In the public database NCBI Assembly, I have found two genome assemblies for this species, whose IDs are SP_G and UP_Spi. It is not clear to me which of these corresponds to the Illumina-based or the Chromium-based assembly in the manuscript. The authors need to sort out this problem and present their correspondences in a more clear-cut way.
The composition of the two genome assemblies in NCBI Assembly differs particularly in the length of the shortest sequence (200bp vs 1000bp) which can largely affect other length-based metrics, including the N50 scaffold length. I wonder what the authors' policy behind this variable length cut-off was, and also how they describe it in the manuscript. If the authors did not have any coherent policy, they should reconsider this point and revise the manuscript and the genome assemblies in the NCBI database.
Also, in the genome assemblies available at NCBI Assembly, I observed a weird distribution of the lengths of 'N' tracts (stretches of undetermined bases) - they are all round numbers for SP_G, while 'N' tracts with the length of 20 is the majority. I wonder whether the authors noticed these, and think that it is worth reasoning possible causes.
For completeness assessment of the genome assemblies they obtained, the authors used the eukaryote ortholog set as well as the Actinopterygii ortholog set. I wonder why the former was used, instead of the vertebrate or metazoan ortholog set. Also, in describing the numbers of orthologs retrieved by BUSCO, the authors should clearly state which category, namely, complete, fragmented, or missing.
Because Figure 2 seems to completely rely on the tool GenomeScope, the authors should cite its source at least in its legend.

Level of Interest

Please indicate how interesting you found the manuscript: Choose an item.

Quality of Written English

Please indicate the quality of language in the manuscript: Choose an item.

Declaration of Competing Interests

Please complete a declaration of competing interests, considering the following questions:

- Have you in the past five years received reimbursements, fees, funding, or salary from an organisation that may in any way gain or lose financially from the publication of this manuscript, either now or in the future?
- Do you hold any stocks or shares in an organisation that may in any way gain or lose financially from the publication of this manuscript, either now or in the future?
- Do you hold or are you currently applying for any patents relating to the content of the manuscript?
- Have you received reimbursements, fees, funding, or salary from an organization that holds or has applied for patents relating to the content of the manuscript?
- Do you have any other financial competing interests?
- Do you have any non-financial competing interests in relation to this paper?

If you can answer no to all of the above, write 'I declare that I have no competing interests' below. If your reply is yes to any, please give details below.

I declare that I have no competing interests

I agree to the open peer review policy of the journal. I understand that my name will be included on my report to the authors and, if the manuscript is accepted for publication, my named report including any attachments I upload will be posted on the website along with the authors' responses. I agree for my report to be made available under an Open Access Creative Commons CC-BY license (http://creativecommons.org/licenses/by/4.0/). I understand that any comments which I do not wish to be included in my named report can be included as confidential comments to the editors, which will not be published.

I agree to the open peer review policy of the journal

To further support our reviewers, we have joined with Publons, where you can gain additional credit to further highlight your hard work (see: https://publons.com/journal/530/gigascience). On publication of this paper, your review will be automatically added to Publons, you can then choose whether or not to claim your Publons credit. I understand this statement.

Yes Choose an item.
